# A Literature Review: Geometric Methods and Their Applications in Human-Related Analysis

**DOI:** 10.3390/s19122809

**Published:** 2019-06-23

**Authors:** Wenjuan Gong, Bin Zhang, Chaoqi Wang, Hanbing Yue, Chuantao Li, Linjie Xing, Yu Qiao, Weishan Zhang, Faming Gong

**Affiliations:** 1 The College of Computer Science and Communication Engineering, China University of Petroleum (East China), Qingdao 257061, China; wenjuangong@upc.edu.cn (W.G.); wcq86451612@163.com (C.W.); iceyueHB@163.com (H.Y.); china_lichuantao@163.com (C.L.); zhangws@upc.edu.cn (W.Z.); gfaming@163.com (F.G.); 2 The Beijing University of Posts and Telecommunications, Beijing 100876, China; fealie28@163.com; 3 Key Laboratory of Human-Machine Intelligence-Synergy Systems, Shenzhen Institutes of Advanced Technology, Chinese Academy of Sciences, Shenzhen 518055, China; csljxing@gmail.com

**Keywords:** geometric methods, human action recognition, human pose estimation, human body shape analysis

## Abstract

Geometric features, such as the topological and manifold properties, are utilized to extract geometric properties. Geometric methods that exploit the applications of geometrics, e.g., geometric features, are widely used in computer graphics and computer vision problems. This review presents a literature review on geometric concepts, geometric methods, and their applications in human-related analysis, e.g., human shape analysis, human pose analysis, and human action analysis. This review proposes to categorize geometric methods based on the scope of the geometric properties that are extracted: object-oriented geometric methods, feature-oriented geometric methods, and routine-based geometric methods. Considering the broad applications of deep learning methods, this review also studies geometric deep learning, which has recently become a popular topic of research. Validation datasets are collected, and method performances are collected and compared. Finally, research trends and possible research topics are discussed.

## 1. Introduction

With the emergence of low-cost RGB-D cameras, human bodies can be digitized at a lower cost [1,2,3], and their actions can also be easily captured [4,5]. In 3D spaces (for point cloud models or meshes), studying the geometric attributes becomes natural. The geometric attributes (for example, the number of holes and the geometric adjacency of objects) of data are extracted, and methods for studying geometric attributes are proposed.

The notion of “geometric methods” is used in this review and refers to methods that study the geometric attributes of data, methods with geometric constraints, or generalized methods with spatial or temporal information. When dealing with continuous 3D models, certain geometries, like topology, Riemann manifold, conformal geometry, etc., are better choices. They are capable of describing the properties of the geometric object from the perspective of the geometric object. In a Euclidean space, the global coordinates are cumbersome for describing attributes along the object surface. For example, the geodesic distance is a better description for two points on a geometric object than the Euclidean distance; the geodesic distance is from the perspective of the points on the surface, and it considers the distance one point needs to traverse on the surface.

Based on the scope of geometric properties that are extracted and the way in which geometric properties are encoded, the review proposes to classify geometric methods into: object-oriented geometric methods, in which the geometric properties of the object surfaces are explored, feature-oriented geometric methods, in which the object features are extracted and the geometric properties of the feature space are explored, and routine-based geometric methods, in which geometric information is extracted following certain routines. For the first category, mathematical concepts and theorems are directly developed and applied. For the second category, geometric theorems can also be developed and applied, but under most circumstances, well-developed geometric concepts and theorems are utilized instead.

Geometric methods are advantageous in several aspects. In many application domains, the data reside on a manifold, e.g., the Grassmannian manifold [6,7], the hypersphere [8,9], or the manifold of symmetric positive definite (SPD) matrices [10,11,12]. Furthermore, using geometric methods results in the concise representation of data. For example, a sphere in a 3D Euclidean space is represented as a 2D geometry in the topology. Furthermore, geometric methods, such as Lie algebra, provide semantic meaning to data operations. For example, adding or subtracting two human body poses denoted by joint positions or limb angles in the Euclidean space has no meaning, while an addition in a Lie group results in a semantically-meaningful pose.

Comparatively, Euclidean distance is not suitable for certain computations, for example, comparing temporal sequences, which is critical for automatic video processing. It is not easy to discriminate sequences among classes. Some methods align the data before using a Euclidean metric, e.g., through dynamic time warping (DTW) [13], specialized kernels or a Fourier hierarchical pyramid [14]; other methods transform the data before using them, e.g., covariance features [15]. None of these methods consider the implicit dynamics of the sequences and the lower dimensional space where the features lie. Geometric spaces provide a possibility for solving this problem.

In this review, attributes and theories in non-Euclidean geometric spaces are explored. These geometric methods and their applications in human-related analysis are collected and studied. “Human-related analysis” (HRA) in this review denotes human shape analysis (HSA), human pose-related analysis (HPA), and human action-related analysis (HAA). HSA includes human shape matching, human shape segmentation, etc.; HPA includes human pose estimation, human posture estimation, human pose modeling, etc.; and HAA contains human action recognition, human activity recognition, etc. Geometric methods are effective solutions for human-related analysis.

Geometric methods were initially utilized in shape analysis and surface registration, which involves understanding the relationship between two geometric objects, for example in finding shape correspondences between two objects [16,17,18,19,20]. Furthermore, geometric methods are utilized in object recognition. For example, moduli space [21] provides a geometric solution for 3D face recognition. Other examples include describing properties in a local area instead of on a single point, like functional correspondences [22], or exploring geometric properties through linear algebra representations, like difference operators [23] among 3D shapes. Shape analysis methods for general objects can be generalized to HSA. Geometric methods can also be utilized for computing correlations between the human shape and another human attribute, e.g., between the shape and the age using manifold regression [24]. Notable works on geometric methods for HPA include Lie group representations of human poses [25]. For HAA, exemplary solutions include: methods of localizing humans in images and tracking and analyzing human motion trajectories and methods that directly extract spatial, temporal, or spatiotemporal patterns from image sequences [26,27].

Recently, many researchers focus on dealing with geometric data using deep learning methods. This research area is named as geometric deep learning and it attempts to generalize deep learning methods to non-Euclidean structured data such as graphs and manifolds. There are mainly two streams of methods: extrinsic methods and intrinsic methods. Extrinsic methods treat 3D data similar as 2D data but with one more dimension. One example of extrinsic methods is volumetric CNNs [28]. This representation is not invariant to deformations. In an intrinsic representation, the filter considers local geometric properties and is applied along the object surface. There are also methods that extend deep learning to manifolds through embedding. Since there is not a canonical embedding for a general manifold, the researchers in [11,12,29,30] proposed solutions for the special case of Riemannian manifolds.

Despite the wide applications of geometric methods especially in HRA, there are few literature reviews. Related works are studied extensively in this literature review. Because geometric concepts require math foundations, mathematical knowledge of geometric spaces, such as basic concepts, geometric properties, and geometric measurements, are firstly introduced. The contributions of this paper include:

- Geometric methods and their applications in human-related analysis are extensively studied.
- Geometric methods are studied based on the scope in which they are applied, and we classify them into: feature-oriented geometric methods, object-oriented geometric methods, and routine-based geometric methods.
- Geometric methods and their performances on standard datasets are collected so that researchers who are interested in this topic can identify the state of the art.

**Figure 1:**
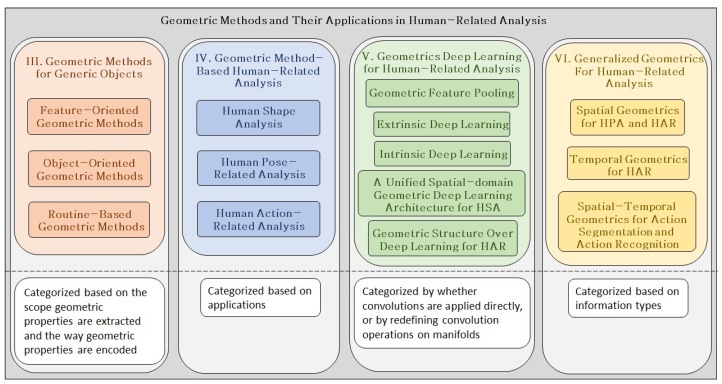
Overall view of this paper. This review is mainly composed of four modules: geometric methods for generic objects; geometric method-based human-related analysis; geometric deep learning for human-related analysis; and generalized geometrics for human-related analysis. Each module has its subsections, each of which is a class of methods based on its categorization standards. HSA, human shape analysis.

The remainder of the paper is organized as follows. Section 2 introduces basic geometric concepts. Section 3 explores variant types of geometric methods. Section 4 explores specific geometric methods for human-related analysis. Section 5 introduces deep learning-based geometric methods. Section 6 studies generalized geometrics for human-related analysis. Section 7 collects a validation dataset for human-related analysis. Section 8 compares the performances of related works. Section 9 concludes the review and discusses future research trends. Figure 1 presents an overall view of the paper.

## 2. Basic Geometric Concepts

**Figure 2:**
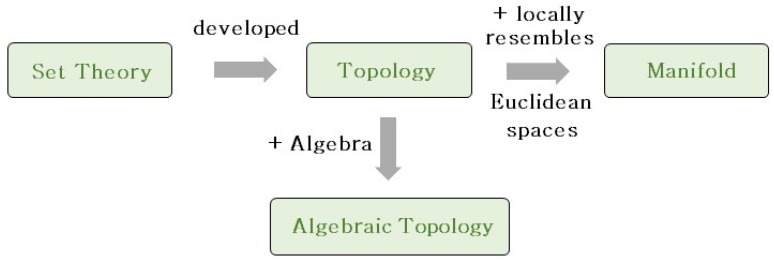
Main components of Section 2. This section is composed of four modules: set theory concepts; topology concepts developed from set theory; algebraic topology concepts (topology plus algebra); and manifold concepts (a topology that locally resembles Euclidean spaces).

In this section, important concepts in the topology and manifolds that are widely used in geometric methods are introduced. Concepts of manifolds are usually difficult to conceive of and can be defined in various ways. This review selects among the different definitions of each concept and chooses the one that is easier to understand. It is more conceivable to consider topological concepts as being developed from set theories, so set theories are firstly introduced. Many concepts of manifolds are developed based on topological concepts, so manifold concepts are introduced following topological concepts. Figure 2 shows the components of this section. Before presenting detailed definitions, the mathematical symbols are listed below.

### 2.1. Set Theory Concepts

An easier way to interpret topology is to consider topological concepts to be developed from set theories. Selected concepts from set theories are introduced in this section. Other concepts, like equivalence relation, equivalence class, and covering, are introduced in the Appendix A.

#### 2.1.1. Metric

A metric or distance function on a set *X* is a real-valued function *d* defined on the Cartesian product X×X such that for all x,y,z∈X:

- d(x,y)≤0 with equality iff x=y.
- d(x,y)=d(y,x).
- d(x,y)+d(y,z)≤d(x,z).

#### 2.1.2. Quotient Vector Space

The quotient of a vector space by a subspace can be defined based on the equivalence class. Let *V* be a vector space over a field *k* and W⊆V be a subspace. An equivalence relation ${∼}_{W}$ on *V* can be denoted by $v{∼}_{W}v^{\prime }$ if and only if $v-v^{\prime }\in W$, where ${∼}_{W}$ is an equivalence relation. The quotient is denoted by *V*/${∼}_{W}$, and *V*/${∼}_{W}$ is itself a vector space over *k*, with the addition and scalar multiplication rules satisfying $\left[v\right]+\left[v^{\prime }\right]=\left[v+v^{\prime }\right]$ and κ[v]=[κv]. *V*/${∼}_{W}$ can also be denoted by *V*/*W*, which is referred to as the quotient space of *V* by *W*.

### 2.2. Topological Concepts

Topology is independent of any particular coordinate representation, while objects’ representation in Euclidean spaces is certain. For example, every point in a three-dimensional Euclidean space is determined by three coordinates, while in topology, a global coordinate system does not exist. The self perspective is the essence of the conciseness in topological representations. It retains more general features, like the number of holes in the geometry while ignoring some fine details, like the distance functions. Specifically, topological properties of a shape are invariant under certain deformations: they do not change if the shape is stretched or compressed, but change under other deformations, like “tearing” or “adjoining”. Topological concepts are selectively introduced in this section. Please refer to the Appendix for definitions of closed sets, the interior and closure of a set, limit points, continuous functions, quotient maps, Hausdorff space, and metrics.

#### 2.2.1. Topology

Here, the geometric view of the topology developed from surfaces and neighborhoods is adopted. According to [31], for each point *x* of a set *X*, the neighborhoods of *x* are a non-empty collection of subsets of *X* and satisfy four axioms:

- *x* lies in each of its neighborhoods.
- The intersection of two neighborhoods of *x* is a neighborhood of *x*.
- If *N* is a neighborhood of *x* and if *U* is a subset of *X* that contains *N*, then *U* is a neighborhood of *x*.
- If *N* is a neighborhood of *x* and if $\overset{\circ }{N}$ denotes the set {z∈N|Nisaneighborhoodofz}, then $\overset{\circ }{N}$ is a neighborhood of *x* (the set $\overset{\circ }{N}$ is called the interior of *N*).

The assignment of a collection of neighborhoods is called a topology on the set *X*. A topology defined with neighborhoods is easy to conceive of, but hard to work with. On the contrary, the topology based on openness is defined. A subset *O* of *X* is open if it is a neighborhood of each of its points. A topological space is then a set *X* together with a collection of open subsets Ω that satisfies the four conditions:

- The empty set ∅ is in Ω.
- *X* is in Ω.
- The intersection of a finite number of sets in Ω is also in Ω.
- The union of an arbitrary number of sets in Ω is also in Ω.

#### 2.2.2. Homeomorphism

**Figure 3:**
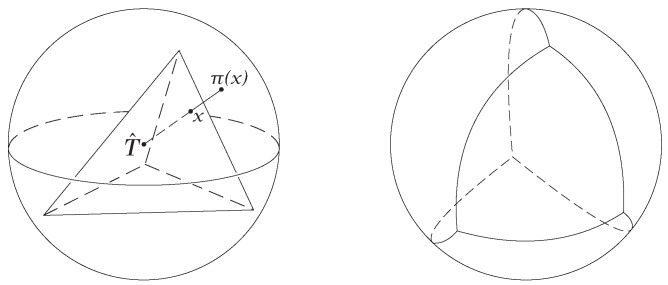
Radial projection from a tetrahedron *T* onto a sphere with center $\hat{T}$. An example is shown as follows: a point *x* on a surface of the tetrahedron projected onto its corresponding point π(x) on the sphere with the radial projection function π.

A function h:X→Y is called a homeomorphism if it is one-one onto continuous and has a continuous inverse. When such a function exists, *X* and *Y* are called homeomorphic (or topologically equivalent) spaces. Figure 3 shows a homeomorphism between a sphere and a tetrahedron. The illustration shows a regular tetrahedron *T* projected onto a sphere with center $\hat{T}$ using radial projections from the center.

#### 2.2.3. Quotient Space

A quotient space is a set together with a topology. If *X* is a space and *A* is a set and if p:X→A is a surjective map, then there exists exactly one topology Ω on *A* relative to which *p* is a quotient map, and it is called the quotient topology induced by *p*.

**Figure 4:**
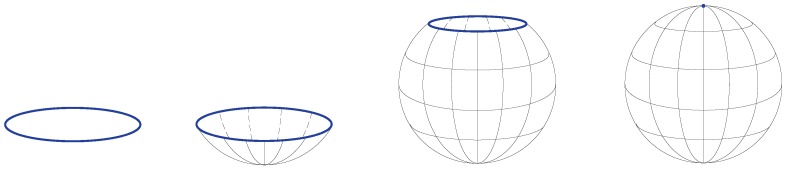
An example of creating a quotient space by gluing. Gluing the boundary of a circle onto a single point. The two-sphere $S^{2}$ is obtained by gluing the circle $S^{1}$ to a single point.

A quotient space (also called an identification space) is, intuitively speaking, the result of identifying or “gluing together” certain points of a given topological space. Figure 4 shows an example of obtaining the two-sphere $S^{2}$ by gluing the circle $S^{1}$ to a single point.

### 2.3. Algebraic Topology Concepts

Algebraic topology combines algebra with geometry by defining algebraic operations on geometric objects. The fundamental idea of algebraic topology is to develop methods for distinguishing between two topological spaces or two maps. The topological group is introduced. Please refer to the Appendix for the definitions of orbit space, homotopy, the fundamental group, and homology.

A topological group is a topological space with a binary operation and the inverse map, both being continuous. For example, *G* is a topological group if the multiplication map (g,h)↦gh from G×G to *G* and the inverse map $g\mapsto g^{-1}$ from *G* to *G* are both continuous. One extremely useful topology group is the general linear group. For example, the general linear group over R, denoted by GL(n,R), is the group of n×n invertible matrices with real entries.

### 2.4. Manifold Concepts

A manifold is both a Hausdorff space and a topological space that locally homeomorphic to the Euclidean space, that is, we can find a continuous bijective invertible mapping between a local area on the manifold and a local area in the Euclidean space. Furthermore, analysis can be carried out by imposing smooth structures on a manifold (similar as a differential Euclidean space). It is not sufficient to develop analysis on the manifold, but with certain methods (like parallel transport), tangent spaces at different points on the manifold are related. Essential concepts in the manifold are introduced in this section. Please refer to the Appendix for definitions of atlas, smooth manifold, section, vector bundle, fiber bundle, the tangent bundle of a vector bundle, vertical bundle, vector bundle homomorphism, vector bundle isomorphism, and connection.

#### 2.4.1. Topological Manifold

Assume that M is a topological space; M is a topological manifold of dimension *n* if it has the following properties:

- M is a Hausdorff space.
- M is second countable: there exists a countable basis for the topology of M.
- M is locally Euclidean of dimension *n*: for each p∈M, we can find an open set U∈M containing *p*, an open set $U^{\prime }\in R^{n}$, and a homeomorphism $\varphi :U\to U^{\prime }$ (i.e., a continuous bijective map with the continuous inverse).

#### 2.4.2. Chart

**Figure 5:**
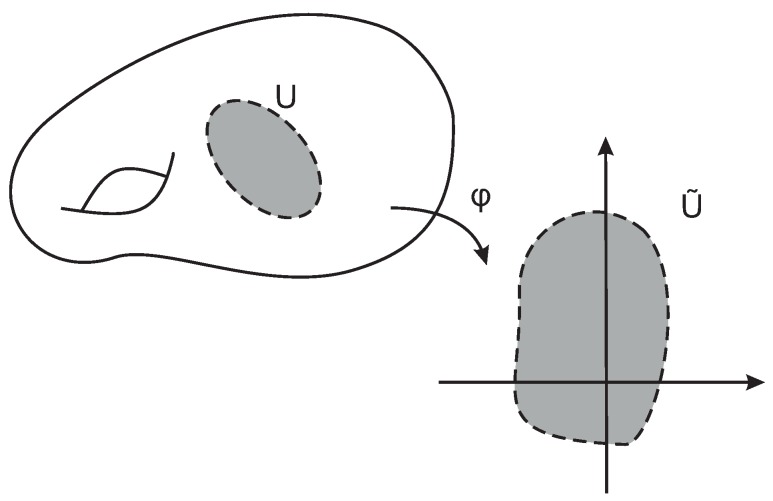
An example of a coordinate chart. The figure illustrates an example of a coordinate chart from *U* to $\tilde{U}$.

A chart, or a coordinate chart, on a manifold M is a pair (U,φ), where *U* is an open subset of M and $\varphi :U\to \tilde{U}$ is a homeomorphism from *U* to an open subset $\tilde{U}=\varphi \left(U\right)\in R^{n}$. Figure 5 illustrates an example of a coordinate chart.

Let M be a topological *n*-manifold. If (U,φ) and (V,ψ) are two charts such that U∩V=⌀, then the composite map $\psi \circ \varphi^{-1}:\varphi \left(U\cap V\right)\to \psi \left(U\cap V\right)$, also called the transition map from φ to ψ, is a composition of homeomorphisms and is a homeomorphism.

Two charts, (U,φ) and (V,ψ), are said to be smoothly compatible if either U∩V=⌀ or the transition map $\psi \circ \varphi^{-1}$ is a diffeomorphism.

#### 2.4.3. Tangent Space/Tangent Bundle

**Figure 6:**
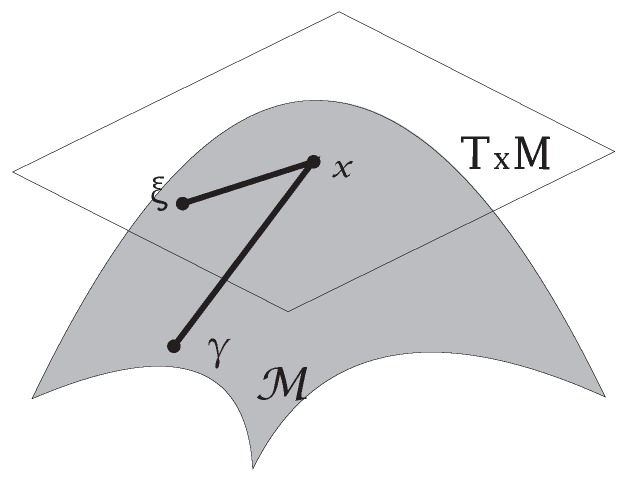
Illustration of a tangent space. $T_{x}M$ is the tangent space of the manifold M at point *x*.

Let M be a smooth manifold, p∈M and $M_{p}$ be the set of all smooth real-valued functions, each of which is defined on some open neighborhood of *p*. A tangent vector to M at *p* is a map $v:M_{p}\to R$ such that:

- v(λf+μg)=λv(f)+μv(g).
- v(fg)=v(f)g(p)+f(p)v(g).

for all $f,g\in M_{p}$, λ,μ∈R. The set of all tangent vectors to M at *p* is denoted by $T_{p}M$. It is called the tangent space to M at *p*. Figure 6 shows an exemplary tangent space.

**Figure 7:**
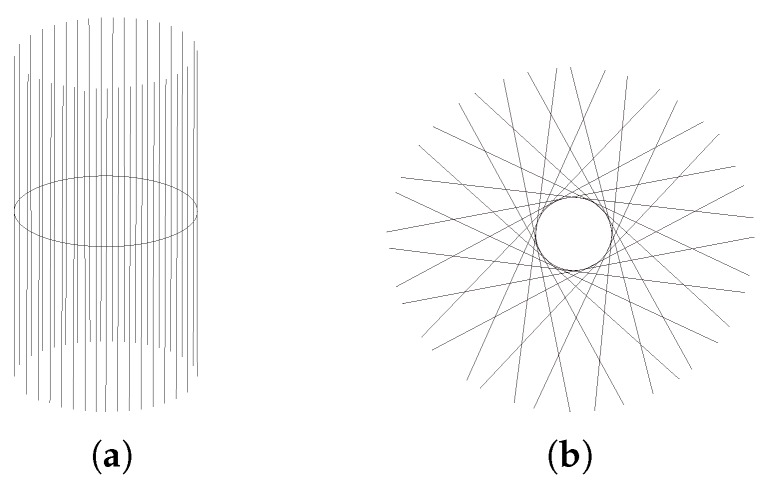
Illustration of a tangent bundle of a manifold. The figure illustrates the tangent bundle of a circle (**a**) viewed from the side and (**b**) viewed from the top or bottom.

The tangent bundle of a manifold M is defined as the disjoint union of all tangent spaces to points of M: $TM=\underset{x\in M}{⋃}T_{x}M$. Figure 7 shows an example of a tangent bundle of a circle. The figure illustrates the tangent bundle of a circle viewed from the side and from the top or bottom. Exemplary tangent spaces and their intersections with the circle are shown.

#### 2.4.4. Parallel Transport

**Figure 8:**
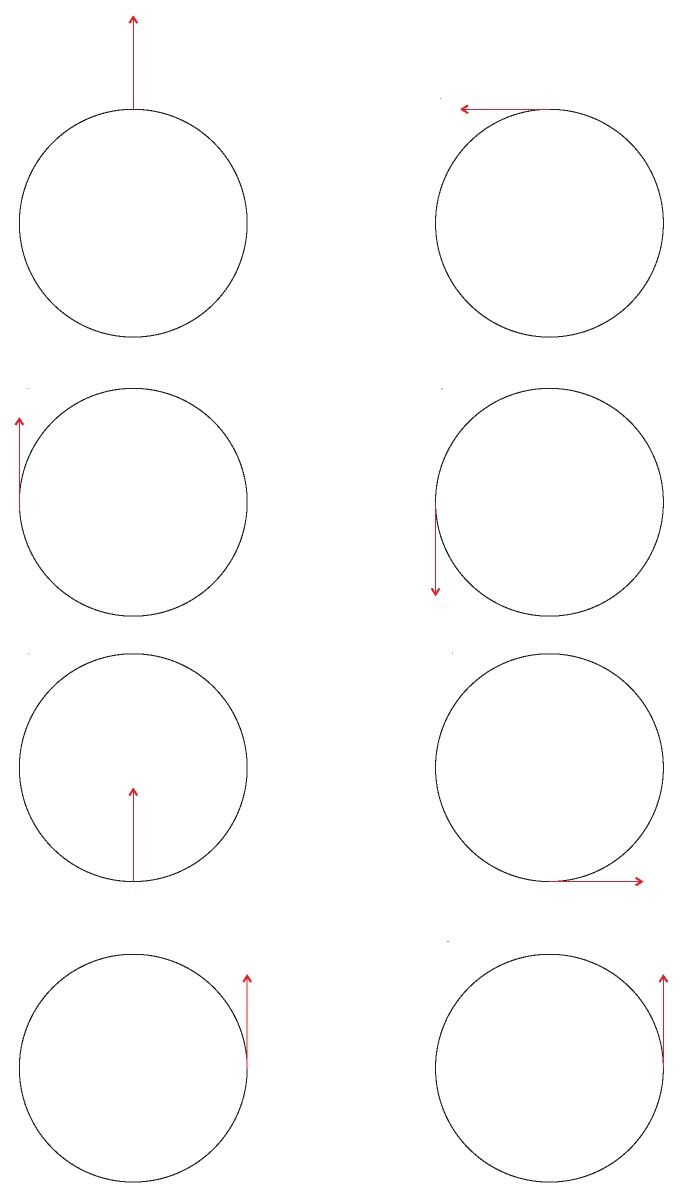
Examples of parallel transports. The figure illustrates two examples of parallel transports under Levi–Civita connections on four sampling positions. The transport on the left side is given by the metric $ds^{2}=dr^{2}+r^{2}d\theta^{2}$. The transport on the right side is given by the metric $ds^{2}=dr^{2}+d\theta^{2}$.

Let M be a smooth manifold with a vector bundle connection ∇; let c:I→M be a differentiable curve from an interval *I* into M; and let $V_{0}\in T_{c(t_{0})}M$ be a vector tangent to M at $c(t_{0})$ for some $t_{0}\in I$. A vector field *V* is said to be a parallel transport of $V_{0}$ along *c* provided that V(t) (t∈I) is a vector field for which $V\left(t_{0}\right)=V_{0}$. The notion of a parallel transport on a manifold M clarifies the idea of translating a vector field *V* along a differentiable curve to attain a new vector field $V^{\prime }$, which is parallel to *V*. Figure 8 shows an illustration of parallel transports under Levi–Civita connections. A Levi–Civita connection is a torsion-free metric connection preserving a given (pseudo-)Riemannian metric.

### 2.5. Lie Group and Lie Algebra

A Lie group is a group *G* that is also an analytic manifold such that for σ,τ∈G, the mapping $\left(\sigma ,\tau \right)\to \sigma \tau^{-1}$ of the product manifold G×G into *G* is analytic. Lie algebra is a vector space g over a field *F* with an operation [·,·]:g×g→g, which we call a Lie bracket, such that the following axioms are satisfied:

- Bilinearity: [ax+by,z]=a[x,z]+b[y,z], [z,ax+by]=a[z,x]+b[z,y] for all scalars *a*, *b* in *F*, and all elements *x*, *y*, *z* in g.
- Skew-symmetry or alternativity: [x,x]=0, which implies [x,y]=-[y,x] for all x,y∈g.
- Jacobi Identity: [x,[y,z]]+[y,[z,x]]+[z,[x,y]]=0.

## 3. Geometric Methods for Generic Objects

In this section, various geometric methods are introduced. Reviewed methods are categorized based on the scope of the geometric properties that are extracted and the way in which the geometric properties are encoded. Some methods encode geometric attributes in features (see Section 3.1); some methods utilize concepts and theories from the topology and manifold and extract geometric properties on objects (see Section 3.2); and some methods extract geometric properties following certain procedures and denote the objects with structured representations, e.g., graph structures (see Section 3.3). There are also methods belonging to multiple categories. For example, the positive definite manifold-based methods in Section 3.1.1 belong to “feature-oriented geometric methods”, and they also belong to “object-oriented geometric methods”. This review selects a logically more appropriate categorization in the case mentioned above. In the following section, geometric methods are studied based on this method of categorization.

Other methods to incorporate geometric information, like regression-based methods [32,33,34,35], manifold diffeomorphisms [36], and others, are also utilized in applications like image processing. These methods are working on 2D objects and are difficult to extend to human-related analysis on 3D data, so they are not elaborated in this review.

### 3.1. Feature-Oriented Geometric Methods

A feature space is the space where an object is projected as a feature point. This section explores the geometric properties of the parameter spaces. Utilizing geometrics in a feature space can be implemented through exploiting neighboring properties of feature points, or through studying geometric attributes and geometric properties in the space.

#### 3.1.1. Distance-Based Methods

Similarities among features extracted from the raw data can be calculated. Distances between sample pairs are extracted and are used to denote geometric attributes. Distances are constructed using similarity measures. The authors in [12] generalized from the case of vector space inputs to the case of a manifold. Distances on manifolds were calculated as geodesic distances between the data [37].

#### 3.1.2. Positive Definite Manifold-Based Methods

Covariance matrices are used to capture representative features [38]. Covariance matrices describe the correlatoin between sampled data points. They are positive definite (PD) matrices and lie on PD manifolds. Temporal sequences are also capable of being embedded in the PD manifold. For example, the authors in [15] built a temporal hierarchy of covariance descriptors for human action classification. Works on computing distances on the PD manifold include [39,40,41,42].

To analyze covariance descriptors, Euclidean geometry is often not appropriate; thus, methods using non-Euclidean metrics have been proposed, e.g., [42,43]. In particular, Gram and Hankel matrices [44,45] and Bregman divergences [29,38,46,47,48] have been successfully applied in a number of covariance descriptor-based applications. Methods considering dynamic information have also been proposed [44,45], in which dynamic information is denoted with Hankel matrices and sequences are compared using the Hankelet subspaces angle. Other examples include [49], in which the authors extended a locally aggregated descriptor (VLAD) to Riemannian manifolds.

In the special case of infinite dimensions, the authors in [50] extended covariance matrices into a Hilbert space.

#### 3.1.3. Kernels over a Manifold

Kernels provide mathematical formulations for covariance matrices. The applications of this type of method include dictionary learning and sparse coding [29,30,51].

Usually, kernels over a manifold are implemented over the Riemannian manifold because the original manifold is required to have distance measures. A Riemannian metric on a manifold *M* is a smoothly-varying inner product 〈·,·〉 on the tangent space $T_{x}M$ at each point x∈M. A Riemannian manifold is a manifold equipped with a Riemannian metric. Some works embed Riemannian manifolds into the reproducing kernel Hilbert space (RKHS). RKHS is a linear space, so it is simple and effective representation. There are also other types of kernels, for example the geodesic exponential kernel in [52], which provides a kernel-based solution for the general Riemannian manifolds.

#### 3.1.4. Moduli Space

For the specific task of classification, moduli space is a natural solution. Moduli spaces can be thought of as geometric solutions to geometric classification problems. Such spaces are the space of equivalence classes of complex structures, where two complex structures are deemed “the same” if they are equivalent by conformal mapping [53]. Two equivalent objects may look very different; but in a moduli space, equivalent objects have the same description, while inequivalent objects have different descriptions.

### 3.2. Object-Oriented Geometric Methods

In object-oriented methods, the geometric attributes of an object are extracted and studied.

#### 3.2.1. Tangent Space-Based Methods

**Figure 9:**
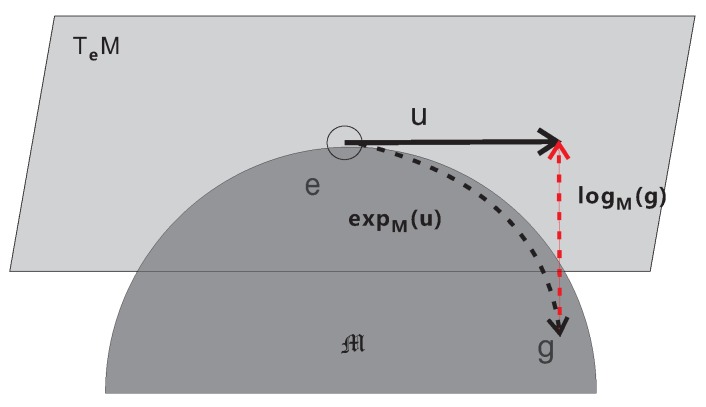
Illustration of the exponential and the logarithmic maps. The example point of *g* on the manifold M is mapped to a point on the tangent plane $T_{e}M$ using a logarithmic map $Log_{M}\left(g\right)$. The exponential map $exp_{M}\left(u\right)$ is the reverse of the logarithmic map.

Tangent spaces (defined in Section 2.4.3) are associated with each point on a manifold. Some of the tangent space-based methods utilize mappings between the tangent space of the manifold and the manifold. An exponential map is a map from the tangent bundle of the manifold to the manifold. In addition, a logarithmic map is its reverse map. The exponential and logarithmic maps are illustrated in Figure 9. The authors in [12] used the Riemannian exponential and logarithmic maps to define a sparse representation on Riemannian manifolds. The formulation is a generalization of the linear sparsity condition to manifolds.

#### 3.2.2. Conformal Geometry-Based Methods

Computational conformal geometry is an interdisciplinary field combining computing and conformal geometry. A conformal mapping is an angle-preserving mapping, and computational conformal geometry designs its algorithms in computing. The authors in [53] presented a thorough description of the theoretical foundations, as well as the practical algorithms of computational conformal geometry. A widely-used application of conformal geometry is in matching two object models. For example, the authors in [16] utilized it to find shape correspondences between two objects. It conformally maps the interior of an *n*-gon *P* bijectively to that of another *n*-gon *Q*. This mapping can be utilized to embed 3D meshes onto a 2D plane. However, when this map is extended to the boundary, it does not necessarily map the vertices of *P* to those of *Q*. For many applications, it is important to identify the “best” vertex-preserving mapping between two polygons, i.e., one that minimizes the maximum angle distortion. It can be considered as conformal geometric methods implemented in a greedy way. Such maps exist, are unique, and are known as extremal quasiconformal maps or Teichmüller maps.

#### 3.2.3. Principal Geodesic Analysis

Principal geodesic analysis (PGA) is an extension of principal component analysis (PCA) to manifolds. PGA has applications in shape analysis [54], and probabilistic PGA was utilized [55] to solve human activity recognition.

Since the objective function in the PGA algorithm is highly non-linear and generally difficult to solve efficiently, researchers who first introduced PGA [56] proposed a linear approximation. Exact computation can also be obtained under certain constraints. For example, the authors in [57] presented an exact computation of the PGA of data on the rotation group SO(3). For constrained manifolds, like the constant curvature Riemannian manifolds in [58], optimization in PGA could be computed efficiently. The authors in [59] also proposed an exact PGA computation method without any linearization for data with a large variance.

### 3.3. Routine-Based Geometric Methods

Following certain routines, geometric information can also be encoded. Reducing representation dimensions, representing objects with a graph, and topological data analysis are all utilized to encode geometric information.

#### 3.3.1. Dimension Reduction-Based Methods

Dimension-reduced representations (also called embeddings) are utilized to study feature space properties [60]. Considering the geometric properties of the feature representation, some non-linear dimension reduction algorithms have been utilized, e.g., the Laplacian eigenmaps (LE) framework, which recovers the low-rank structure of the manifold in a projected space. Laplacian eigenmaps [61] use graphs to find the embedding of the data in a low-dimensional space.

Furthermore, additional structures from low-dimensional data can be utilized as prior knowledge to enhance the representability of the models [62,63,64]. Discrete graphs are also utilized to incorporate data manifold information into the dimensionality reduction framework [65,66,67,68,69,70,71,72,73].

#### 3.3.2. Graph-Based Methods

Graphs are concise representations for structural data. Graphs consist of units and connections. Units are connected if certain criteria are met. One wide application of graphs is to construct a mesh model from point clouds, in which units are connected if the distance between a pair is below a threshold. After graphs are constructed, clustering is usually utilized to explore the geometrically-adjacent attributes. One method of clustering a point cloud is single linkage clustering and its extensions [74,75]. In the single-linkage clustering methods, a graph is constructed with the vertex set as the set of points in the cloud and the edges as point connections if their distance is less than a threshold.

Under the assumption that high-dimensional data samples lie on or close to a smooth low-dimensional manifold, and the manifold can be approximated discretely as a graph, graphs can also be utilized to describe the low-dimensional intrinsic structure of the high-dimensional data. The emerging field of signal processing on graphs also facilitates the graph representation of signals [76].

#### 3.3.3. Topological Data Analysis

In a broader perspective, topological data analysis (TDA) is an approach to analyzing data using topological methods [77,78] and is closely related to persistent homology, an adaptation of homology (defined in Appendix A.3.4) to point cloud data.

**Figure 10:**
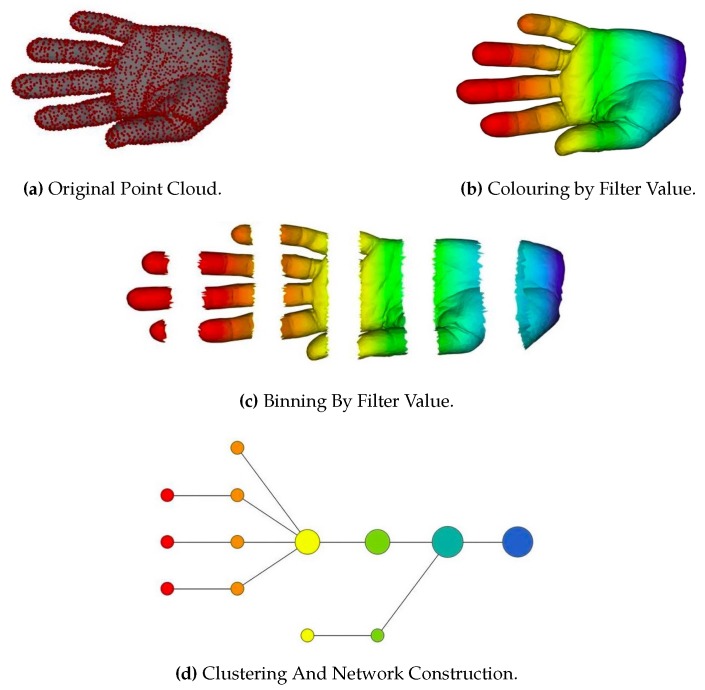
Illustration of a topological data analysis (TDA) pipeline. (**a**) A 3D object (hand) represented as a point cloud. (**b**) A filter value is applied to the point cloud, and the object is now colored by the values of the filter functions. (**c**) The data points are binned into overlapping groups. (**d**) Each bin is clustered and a center of the cluster is calculated, and a network is built by connecting the cluster center sequentially. The figure is originally from [79].

The TDA mentioned here refers to certain procedures for extracting topological properties from point cloud data. For example, the authors in [79] analyzed the geometric adjacency properties of an object and represented the object as a graph composed of nodes denoting key parts of the object. The graph considered the topological properties of the object. Topological properties are denoted by a topological network, i.e., a collection of nodes and a collection of edges connecting some of the nodes. Figure 10 shows the pipeline of the proposed method. TDA summarizes the data in a way that keep its global structure and local details to some degree, which is missing in other analysis methods, such as principal component analysis (PCA), multidimensional scaling (MDS), and cluster analysis.

## 4. Geometric Method-Based Human-Related Analysis

For articulated objects, like human bodies, extrinsic properties are not capable of describing their intrinsic properties, like shapes and symmetric properties. Although suffering from topological noise, isometry-preserving properties are widely used for human-related analysis, e.g., the methods from [80,81,82,83,84,85] are utilized for human shape analysis, and the method from [86] is utilized for human shape recognition. Geometric methods are also isometry-preserving methods. In this section, geometric methods applied in human-related analysis are explored. Aiming at various application scenarios, different geometric methods are utilized including those introduced in Section 3. The methods in this section are classified based on the applications. There are also works with literature reviews for specific applications, e.g., mesh segmentation [87], shape analysis [88,89], or shape retrieval [90].

### 4.1. Human Shape Analysis

General shape analysis has a wider scope than HSA. Shape comparisons, computing shape summary statistics, mathematical modeling of shape variations, and shape synthesis are all included in general shape analysis. 3D human shape synthesis is plausible using general shape synthesis methods, and this review concentrates on analyzing the human models instead of editing them, so shape synthesis is not the focus of this review. Shape summary statistics and shape variation modeling-related methods are discussed in the human pose-related analysis subsection. In this section, shape comparisons are discussed.

In computer graphics, object shapes are usually compared through a metric, or the dissimilarity measure. Geodesics are important for computing distances between object samples in representation space (e.g., a shape space) or on the shape surface. Spectral analysis is one widely-used method for measuring 3D human shape geodesics. Spectral analysis is an analysis in terms of eigenvalues (e.g., heat kernel signature-based method in Section 4.1.1), frequency spectrum (e.g., the learned spectral descriptor-based method in Section 4.1.3), etc.

Furthermore, diffusion geometry has been studied and utilized to describe intrinsic geometric properties of objects. In diffusion geometry, the distances between points are denoted in a way so that this is transformed into a metric learning problem, and various kernels are used, including the heat kernel, the wave kernel, etc.

#### 4.1.1. Heat Kernel-Based Methods

The behavior of a quantum particle on the manifold is modeled by the Schrödinger equation:

(1) $$\left(i\Delta_{M}+\frac{\partial }{\partial t}\right)\psi \left(x,t\right)=0,$$

where ψ(x,t) is the function capturing the particle behavior, and $\Delta_{M}$ is the Laplace–Beltrami operator (LBO) of M:

(2) Δ:=div▿,

which is the divergence of the gradient. The divergence is the extent to which some quantity is exiting an infinitesimal region of a space, and the gradient is a multi-variant version of the derivative. LBO is the generalization of the Laplacian on Riemannian manifolds.

Given an initial heat distribution f:M→R, let $H_{t}\left(f\right)$ denote the heat distribution at time *t*: $H_{t}=e^{-t\Delta_{M}}$. The heat kernel is based on the exponential function of the eigenvalues of the LBO [91]: $k_{t}\left(x,y\right):R^{+}\times M\times M\to R$ and satisfies $H_{t}f\left(x\right)=\int_{M}k_{t}\left(x,y\right)f\left(y\right)dy$, where $d_{y}$ is the volume form at y∈M:

(3) $$k_{t}\left(x,y\right)=\sum_{i=0}^{\infty }e^{-\lambda_{i}t}\phi_{i}\left(x\right)\phi_{i}\left(y\right).$$

The heat kernel signature (HKS) [92] is a dense descriptor constructed by considering the diagonal of the heat kernel:

(4) $$k_{t}\left(x,x\right)=\sum_{k\geq 0}e^{-t\lambda_{k}}\phi_{k}^{2}\left(x\right).$$

It is also known as the autodiffusivity function. Additionally, the HKS of dimension *Q* at point *x* is defined by sampling the autodiffusivity function at some fixed times $t_{1},\cdots ,t_{Q}$:

(5) $$f\left(x\right)={\left(k_{t_{1}}\left(x,x\right),\ldots ,k_{t_{Q}}\left(x,x\right)\right)}^{T}.$$

#### 4.1.2. Wave Kernel Signature-Based Methods

The wave kernel signature (WKS) evaluates the probability of a quantum particle being located at a point of a manifold under a certain energy distribution. The probability of finding the particle at point *x* is given by:

(6) $$p\left(x\right)=\lim_{T\to \infty }\int_{0}^{T}{\left|\psi \left(x,t\right)\right|}^{2}dt=\sum_{k\geq 1}\pi^{2}\left(\lambda_{k}\right)\phi_{k}^{2}\left(x\right).$$

The definition depends on the initial frequency distribution π(λ). For example, the authors in [93,94] considered a log-normal frequency distribution $\pi_{\nu }\left(\lambda \right)=\exp\left(\frac{\log\nu -\log\lambda }{2\sigma^{2}}\right)$ with mean frequency ν and standard deviation σ. The *Q*-dimensional wave kernel signature (WKS) is defined as:

(7) $$f\left(x\right)={\left(p_{\nu_{1}}\left(x\right),\ldots ,p_{\nu_{Q}}\left(x\right)\right)}^{T},$$

where $p_{\nu }\left(x\right)$ is the probability Equation (6) corresponding to the initial log-normal frequency distribution with mean frequency ν, and $\nu_{1},\ldots ,\nu_{Q}$ are some logarithmically-sampled frequencies.

#### 4.1.3. Learned Spectral Descriptor-Based Methods

Under the proposition that the descriptor should consider the statistics of the corpus of shapes (for example, thin and fat human models) and those of the class of transformations (such as human pose variations), the authors in [95] proposed a learning scheme for the construction of optimized spectral descriptors and formulated the descriptor in a generic form:

(8) $$f\left(x\right)=\sum_{k\geq 1}\tau \left(\lambda_{k}\right)\phi_{k}^{2}\left(x\right)\approx \tau \left(\lambda_{k}\right)\phi_{k}^{2}\left(x\right),$$

where $\tau \left(\lambda \right)={\left(\tau_{1}\left(\lambda \right),\ldots ,\tau_{Q}\left(\lambda \right)\right)}^{T}$ is a bank of transfer functions acting on the LBO eigenvalues, and the parametric transfer function:

(9) $$\tau_{q}\left(\lambda \right)=\sum_{m=1}^{M}a_{qm}\beta_{m}\left(\lambda \right)$$

is defined in terms of the B-spline basis $\beta_{1}\left(\lambda \right),\ldots ,\beta_{M}\left(\lambda \right)$ and the parametrization coefficients $a_{qm}$(q=1,…,Q,m=1,…,M). Plugging Equation (9) into Equation (8), the $q^{\mathrm{th}}$ component of the spectral descriptor is represented as:

(10) $$f_{q(x)}=\sum_{k\geq 1}\tau_{q}\left(\lambda_{k}\right)\phi_{k}^{2}\left(x\right)=\sum_{m=1}^{M}a_{qm}\underset{g_{m}\left(x\right)}{\underset{︸}{\sum_{k\geq 1}\beta_{m}\left(\lambda_{k}\right)\phi_{k}^{2}\left(x\right)}},$$

where $g\left(x\right)={\left(g_{1}\left(x\right),\ldots ,g_{M}\left(x\right)\right)}^{T}$ is a vector-valued function dependent only on the intrinsic geometry of the shape. Thus, Equation (8) is parametrized by the Q×M matrix $A=(a_{qm})$ and can be written in matrix form as f(x)=Ag(x). The main idea of [95] is to learn the optimal parameters A by minimizing a task-specific loss, which reduces to Mahalanobis-type metric learning.

**Figure 11:**
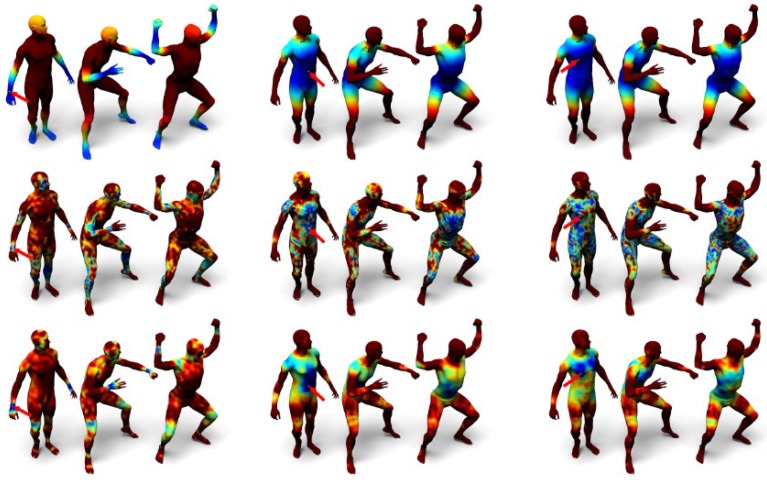
Three kernel-based distance visualized on human models. Visualized distances between the reference point (pointed with red arrows in the first column of each sub-group) and other points on the model. On the left, the reference point is the right writs, in the middle the belly, and on the right the chest. The first row shows the results from the heat kernel, the second row shows the results form the wave kernel, and the third row shows the results of the proposed kernel in [95]. Dark blue shows small distances; red represents large distances. ©2014 IEEE. Reprinted, with permission, from R. Litman, and A. M. Bronstein, Learning Spectral Descriptors for Deformable Shape Correspondence, *in IEEE Trans. Pattern Anal. Mach. Intell.*, 2014, *36*, 170–180.

**Figure 12:**
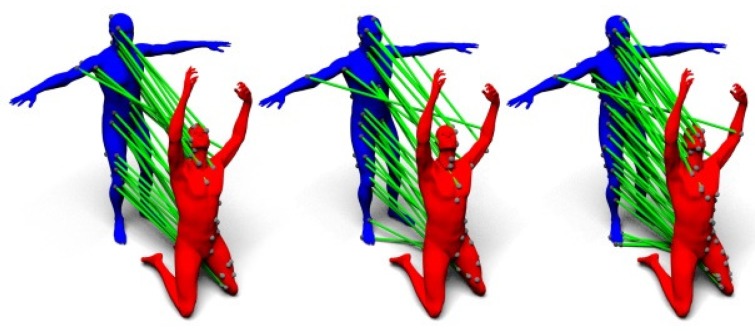
Heat kernel signature (HKS), wave kernel signature (WKS), and learned spectral descriptors for point matching between human models. Correspondences computed on TOSCA shapes with geodesic distance distortion below 10% of the shape diameter using the heat kernel signature, wave kernel signature, and learned spectral descriptor (from left to right) [95]. ©2014 IEEE. Reprinted, with permission, from R. Litman, and A. M. Bronstein, Learning Spectral Descriptors for Deformable Shape Correspondence, *in IEEE Trans. Pattern Anal. Mach. Intell.*, 2014, *36*, 170–180.

Figure 11 visualizes the distances computed from the three kernels mentioned in this section, and Figure 12 shows the computed correspondences between two human models using the three kernels.

### 4.2. Human Pose-Related Analysis

Pose space deformation methods are widely used in human pose morphing. Based on the pose space deformation methods, model reduction has proven useful to increase the performance of static pose-space deformation both with [96,97,98] and without dynamics [99]. Given morphing targets, some works [96] constructed a single pose-independent basis by performing PCA on the sets of bases computed at the underformed configuration. Others obtained the basis by performing PCA on full simulation data [97,98,99]. To accommodate large deformations, the basis can be improved using modal derivatives [100] or linear transformations of the basis [101].

Pose-space subspace methods are utilized in human pose representation to construct reduced-order models with pose-dependent bases [102]. Variant subspace is computed for each representative set and these subspace is further combined into a dynamic system.

In Euclidean space, adding two poses might result in a physically-infeasible pose. Methods for representing 3D human poses with Lie groups have been proposed to solve this problem [25]. Lie group theory provides a semantically meaningful space for adding and subtracting human poses.

### 4.3. Human Action-Related Analysis

Human actions are recognizable from both still images and videos (or image sequences). When dealing with videos (or image sequences), temporal information is beneficial to boost the action recognition accuracy.

#### 4.3.1. Relative 3D Geometry-Based Methods for Human Action Recognition

**Figure 13:**
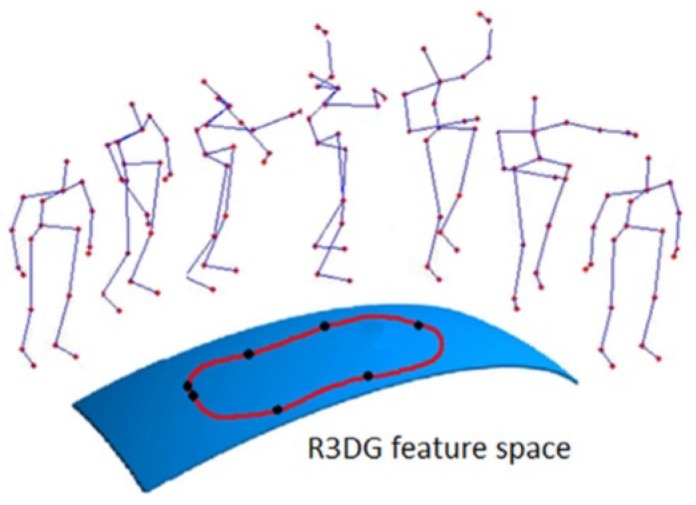
An action trajectory in R3DGfeature space. One point on the action trajectory is an R3DG feature of a pose [103]. Reprinted from Comput. Vis. Image Underst., Vol. 152, R. Vemulapalli, F. Arrate, and R. Chellappa, R3DG features: Relative 3D geometry-based skeletal representations for human action recognition, 155–166, Copyright 2016, with permission from Elsevier.

Many of the skeleton-based approaches for human action recognition use joint locations and joint angles to represent human poses. For example, the authors in [103] introduced a family of skeletal representations for HAR. The family of the proposed features used the relative 3D rotations between various body parts. They were split into two groups: four transformation-based features and two rotation-based features. Using the proposed representations, human actions are modeled as curves in the R3DG feature space (illustrated in Figure 13). Action recognition is then performed by classifying these curves with a combined method of dynamic time warping, Fourier temporal pyramid representation, and support vector machines.

#### 4.3.2. Matrix Embedding for 3D Human Action Recognition

Hankel matrices carry useful invariant properties, e.g., the rank of the Hankel matrix measures the complexity of the underlying dynamics [45]. Despite its advantages, Hankel matrices are not robust against noise. The authors in [104] embedded the sequences into a Riemannian manifold by using positive definite regularized Gram matrices of their Hankelets. Gram matrices inherit the rank and invariance properties of the associated Hankel matrices. Furthermore, Gram matrices are confined to the positive semi-definite (PSD) manifold and capture the underlying geometry better than directly comparing the sequences or Hankel matrices.

#### 4.3.3. Graph-Based Human Action Recognition

Graph-based algorithms have been widely used for action recognition in conventional RGB videos [105,106,107]. Interesting works include graph representations for high-level features. For example, the authors in [108] proposed a graph representation for skeleton-based 3D action recognition. A node of the graph is modeled as a motionlet, which is a semantic part of the trajectory of a joint. The edge is labeled as spatiotemporal relationships between connected motionlets. Constructed graphs are decomposed into substructures called subgraphs, and these subgraphs are compared based on a proposed graph kernel named the subgraph-pattern graph kernel (SPGK). The proposed kernel considers both spatial and temporal information. To circumvent the NP-hard problem of extracting all subgraph patterns from a graph, the authors reformulated the kernel using dynamic programming.

#### 4.3.4. Lie Group-Based Human Action Recognition

Given human skeletal representations in a Lie group, human actions can be represented as curves in this Lie group. The authors in [109] used this type of method. First, a skeletal representation was proposed to explicitly model the 3D geometric relationships between various body parts using rotations and translations in the 3D space. The proposed skeletal representation lies in the Lie group SE(3)×⋯×SE(3), which is a curved manifold. Using the proposed representation, human actions can be modeled as curves in this Lie group. Due to the difficulty of classifying curves in the Lie group, the action curves from the Lie group are mapped to its Lie algebra, which is a vector space. Then, classification is performed with a combined method of dynamic time warping, Fourier temporal pyramid representation, and linear SVM.

The authors in [110] used a similar pipeline of first representing skeletons with Lie groups and then classifying the actions, represented as curves, in Lie groups. Specifically, each skeleton is represented using the relative 3D rotations between various body parts. The skeletal representation is a point in the Lie group SO(3)×⋯×SO(3). Then, using this representation, human actions are modeled as curves in this Lie group. The action curves are mapped onto its Lie algebra by combining the logarithm map with rolling maps, and classification is performed in the Lie algebra.

#### 4.3.5. Dynamic Manifold Warping for Human Action Recognition

For temporal misalignment problems on a manifold, dynamic time warping algorithms are adapted for solving human action recognition problems. For example, the authors in [111] proposed a spatiotemporal manifold (STM) model to analyze human action trajectories with latent spatial structure. Action sequences were aligned with respect to latent parameters, which encoded a path as a point moving on a manifold from a starting point with a parameter value of zero to an ending point with a parameter value of one. In addition, a motion similarity metric was proposed for human action sequences, both in 2D and 3D.

## 5. Geometric Deep Learning for Human-Related Analysis

Deep learning has achieved remarkable performance breakthroughs in speech recognition, natural language processing, and computer vision. In particular, convolutional neural network (CNN) architectures perform well on many image analysis tasks such as classification [112], segmentation [113,114,115], regression [116], and synthesis tasks [117]. A convolution can be thought of as a template matching with filters, and convolution operations on a whole image are carried out by a sliding window procedure. In the case of processing images, one extracts a patch of pixels within a window, correlates it with a template, and moves the window to the next position. Recently, geometric deep learning [118,119] has been the focus of considerable research attention (http://geometricdeeplearning.com/, https://sites.google.com/site/deepgeometry/), while literature reviews on specific applications remain absent.

**Figure 14:**
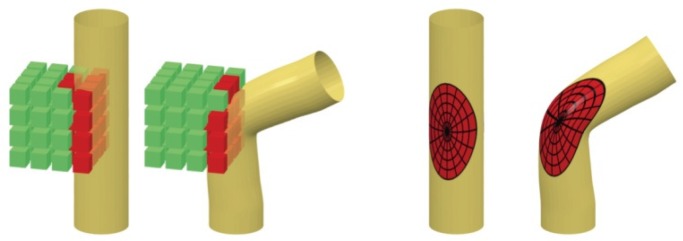
Illustrations of the differences between extrinsic CNN and intrinsic CNN. Intrinsic methods (right) work on the manifold rather than its Euclidean realization. The figure is originally from [120]. Reproduced with permission from Michael Bronstein, NIPS Proceedings; published by Neural Information Processing Systems Foundation, Inc., 2016.

In this section, geometric deep learning methods and their applications in human-related analysis are studied extensively. Based on how geometric information is utilized, by directly applying traditional convolution operations to geometric objects or by redefining convolution operations and traversing methods on manifolds, geometric deep learning is classified into extrinsic deep learning methods and intrinsic deep learning methods. Figure 14 compares these two types of methods implemented with CNN. Extrinsic CNN (the left subfigure in Figure 14) extends the traditional convolution operation from 2D to 3D and does convolution using the 3D templates shown as the cube in the figure. On the contrary, intrinsic CNN (the right subfigure in Figure 14) defines convolution on the manifold, i.e., along the object surface, and the dimensions of the convolution operations can be considered as 2D.

Feature pooling is also an important module in the deep learning architecture, and it is crucial for dimension reduction. Therefore, Section 5.1 introduces feature pooling methods, and the rest of this section explains various ways to define convolutions.

### 5.1. Geometric Feature Pooling

Feature pooling is a key component for reducing representation dimensions. Two prevailing pooling techniques, namely average and max poolings, are not theoretically optimal due to the unrecoverable loss of the spatial information. The authors in [121] proposed generalizing previous pooling methods towards a weighted $\ell_{p}$-norm spatial pooling function tailored for class-specific feature distributions. Specifically, the pooled features are weighted by the image location of a specific visual word. The original method was proposed under the bag of words (BoW) pipeline, but theoretically, it can be adapted to the deep learning architecture.

### 5.2. Extrinsic Deep Learning

Deep CNNs have recently been adapted to process 3D data by generalizing standard 2D convolutions to 3D. These methods of treating geometric data are called extrinsic methods. Their applications include processing 3D geometric shapes, for example, 3D object detection from RGB-D data [122], object classification of point clouds data [123], 3D object local feature matching [124], and 3D deformation flows [125].

#### 5.2.1. Volumetric CNN for Shape Analysis

A natural extension to the classic CNN that processes 2D images is to process 3D data using a volumetric representation and perform 3D convolutions. The authors in [28] presented a 3D deep learning framework for modeling shapes using a voxel representation for 3D object shapes, called ShapeNets. The approach represents a geometric 3D shape as a probabilistic distribution in a voxel grid, and a convolutional deep belief network is used to learn the joint distribution of all voxels. The dataset and the source code are available (http://3DShapeNets.cs.princeton.edu). This generic shape analysis algorithm is applicable to human body models.

#### 5.2.2. Geometric Constrained Extrinsic CNN for Human Shape Analysis

**Figure 15:**
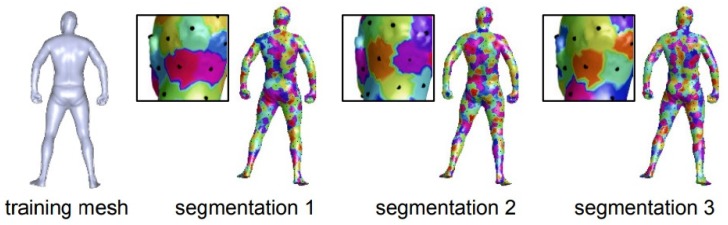
A Training Mesh Example with Its Multiple segmentations. To ensure smooth descriptors, the authors in [126] defined a classification problem for multiple segmentations of the human body. Points on the boundary might be assigned to nearby classes in different segmentation. ©2016 IEEE. Reprinted, with permission, from L. Wei, Q. Huang, D. Ceylan, E. Vouga, and H. Li, Dense Human Body Correspondences Using Convolutional Networks, *in Proceedings of the European Conference on Computer Vision*, Amsterdam, The Netherlands, 11–14 October 2016, 1544–1553.

Instead of adapting the convolution operations in the network, geometric information can also be incorporated through other measures. Traditional classification neural networks tend to separate the surface points lying in different, but nearby classes, which results in ambiguous point categories at the segmentation boundaries. To solve this problem, the authors in [126] proposed smoother feature representations. The CNN network consists of layers of descriptor extractions and a classification layer and removing the classification layer after training leaves the descriptor extraction network. This architecture is widely used for feature extraction. Extracted features are then fused with an ensemble of classification tasks. To ensure descriptor smoothness, the authors proposed randomizing the dense-label generation procedure. Specifically, multiple segmentations of the same person were considered (shown in Figure 15), and a classification problem was introduced for each. The source code and the dataset are available (https://github.com/halimacc/DenseHumanBodyCorrespondences).

### 5.3. Intrinsic Deep Learning

Alternatively, the convolution operations and how the convolution operates over the whole object are redefined on a manifold. This type of methods are called intrinsic methods.

#### 5.3.1. Spatial-Domain Geometric CNN for Human Shape Analysis

**Figure 16:**
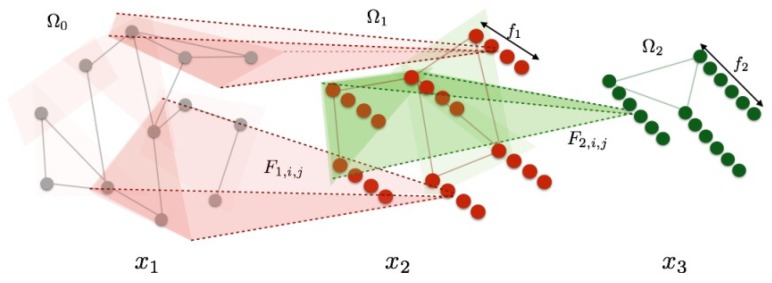
Spatial construction of geometric CNN. K(K = 2 in the example) scales are considered. $\Omega_{k}$ is defined as a partition of $\Omega_{k-1}$ into $d_{k}$ clusters. Each layer of the network transforms a $f_{k-1}$-dimensional signal indexed by $\Omega_{k-1}$ into a $f_{k}$-dimensional signal indexed by $\Omega_{k}$. The figure is originally from [127].

A straightforward method for defining an intrinsic equivalent of a convolution is through the spatial domain. One method is to consider local receptive fields, in which the grid is replaced by a weighted neighborhood. Figure 16 shows an exemplary construction of a spatial-domain geometric CNN.

**Figure 17:**
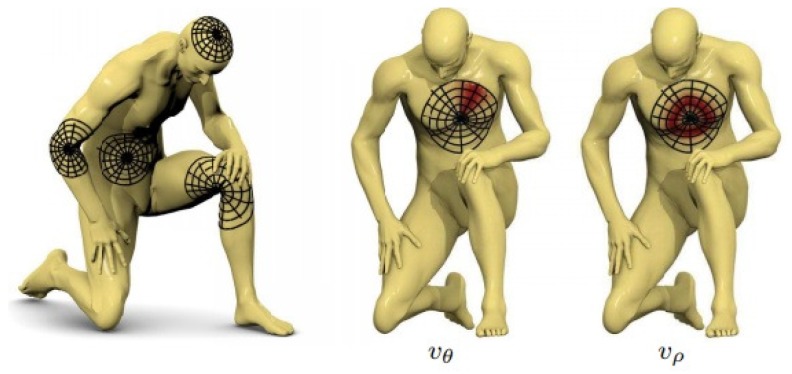
Visualized local geodesic polar coordinates. Left: examples of local geodesic patches, center and right: examples of angular weights and radial weights, $v_{\theta }$ and $v_{\rho }$, respectively (red denotes larger weights) [128]. ©2015 IEEE. Reprinted, with permission, from J. Masci, D. Boscaini, M. M. Bronstein, and P. Vandergheynst, Geodesic Convolutional Neural Networks on Riemannian Manifolds, *in Proceedings of the IEEE Workshop on 3D Representation and Recognition*, Santiago, Chile, 17 December 2015, 832–840.

Another approach utilizes local polar coordinate systems. The authors in [128] defined the patch operator as a combination of Gaussian weights defined on a local polar system of coordinates (shown in Figure 17). After extracting the local geodesic coordinate system, the geodesic patch operator is defined as:

(11) $$\begin{array}{ccc} \left(D(x)f)(\theta ,\rho \right) & = & \int_{X}f\left(x^{\prime }\right)w_{\theta }\left(x,x^{\prime }\right)w_{\rho }\left(x,x^{\prime }\right)dx^{\prime }, \end{array}$$

(12) $$\begin{array}{ccc} w_{\theta }\left(x,x^{\prime }\right) & = & e^{-d_{X}^{2}\left(\Gamma \left(x,\theta \right),x^{\prime }\right)/2\sigma_{\theta }^{2}}, \end{array}$$

(13) $$\begin{array}{ccc} w_{\rho }\left(x,x^{\prime }\right) & = & e^{-{\left(d_{X}\left(x,x^{\prime }\right)-\rho \right)}^{2}/2\sigma_{\rho }^{2}}, \end{array}$$

where $w_{\theta }$ and $w_{\rho }$ are the angular weight and the radial weight, respectively. An angular max pooling was used due to the difficulties of fixing the angular axes at each sampled point, leading to the following definition of the geodesic convolution:

(14) $$\left(f⋆w\right)\left(x\right)=\max_{\begin{array}{c} \Delta \theta \in [0,2\pi ) \end{array}}\int w\left(\theta +\Delta \theta ,\rho \right)\left(D\left(x\right)f\right)\left(\theta ,\rho \right)d\theta d\rho .$$

Furthermore, Fourier transform layers and covariance layers are also defined to transform signals into the frequency domain and inspect the global features from all input dimensions.

#### 5.3.2. Spectral Analysis-Based Intrinsic CNN

Another type of method generalizes the convolution operator with the spectrum analysis. A fundamental result of classical Euclidean signal processing states that the Fourier transform diagonalizes the convolution operator [119]. Then, convolutions may be extended to general manifolds by finding the corresponding basis. In the case of graph representations, the convolution operator can be carried out with the spectrum of its graph Laplacian. For example, in [127], convolution operations are defined as follows: for each layer k=1…K, an input vector $x_{k}$ of size $\left|\Omega \right|\times f_{k-1}$ is transformed into an output $x_{k+1}$ of dimensions $\left|\Omega \right|\times f_{k}$:

(15) $$x_{k+1,j}=h\left(V\sum_{i=1}^{f_{k-1}}F_{k,i,j}V^{T}x_{k,i}\right)\;\left(j=1\ldots f_{k}\right),$$

where $F_{k,i,j}$ is a diagonal matrix, *V* is composed of the eigenvectors of the Laplacian, and *h* is a real-valued non-linear function. In addition, filters with constant spatial support are obtained by choosing specific sampling steps in the spectral domain.

##### Localized Spectral CNN for Human Shape Analysis

One drawback of spectral analysis is the difficulty in the spatial localization. Spectral analysis is global because the basis functions are global. There are studies that specialize in spatial localization through operations on the spectral domain. In [129,130], these operations were achieved through windowed Fourier transform on the spectral domain.

The windowed graph Fourier transform (WGFT) of a signal *f* [129,130] can be defined through the filtering signal *g*:

(16) $$\left(Sf\right)\left(x,k\right):=\langle f,g_{x,k}\rangle ,$$

where $g_{x,k}\left(n\right)$ is a windowed element centered at vertex *x* and frequency *k*:

(17) $$\begin{array}{cc} g_{x,k}\left(n\right):= & \left(M_{k}T_{x}g\right)\left(n\right)\\ = & N\chi_{k}\left(n\right)\sum_{l=0}^{N-1}\hat{g}\left(\lambda_{l}\right)\chi_{l}^{*}\left(x\right)\chi_{l}\left(n\right). \end{array}$$

Then, WGFT can be reformulated as:

(18) $$\left(Sf\right)\left(x,k\right)=\sum_{l=0}^{N-1}{\hat{g}}_{l}\chi_{l}^{*}\left(x\right)\langle f,\chi_{l}\chi_{k}\rangle .$$

The WGFT (Sf)(x,k) filters signal *f* at point *x* at frequency *k* with a window defined by ${\hat{g}}_{l}$.

By collecting its behavior over different frequencies, the content of signal *f* in a local support around *x* is extracted, thus reproducing the window extraction on images. The localized spectral convolution layer can thus be defined as:

(19) $$f_{q}^{out}\left(x\right)=\sum_{p=1}^{P}\sum_{k=1}^{K}w_{q,k,p}\left|\left(Sf_{p}\right)\left(x,k\right)\right|,$$

where $f_{p}$(p=1,…,P) is the input signal, $W=(w_{q,k,p})$ is a Q×K×P tensor representing the learnable weights, and $f_{q}^{out}$(q=1,…,Q) is the output signal.

#### 5.3.3. Heat Diffusion CNN for Human Shape Analysis

The heat diffusion equation is also used for extending traditional CNN to a manifold. Heat diffusion measures heat diffused on a manifold. The heat propagation on a shape *X* is governed by the heat diffusion Equation (1). Given the initial heat distribution a delta function centered on *x*, the heat distribution on *X* after some time *t* is represented by the heat kernel $h_{t}\left(x,\cdot \right)$. The heat kernel, as formulated in Equation (3), is isotropic. Generalized heat diffusion is described by the anisotropic diffusion equation:

(20) $$f_{t}\left(x,t\right)=-{\mathrm{div}}_{X}\left(A\left(x\right)\nabla_{X}f\left(x,t\right)\right),$$

where $\nabla_{X}$ and ${\mathrm{div}}_{X}$ denote the intrinsic gradient and divergence operators and f(x,t) is the temperature at point *x* at time *t*. The thermal conductivity matrix A(x) specifies the heat conductivity properties at each point on shape *X*. The general diffusion model can be utilized for shape analysis [131].

The authors in [120] defined the thermal conductivity matrix as:

(21) $$A_{\alpha \theta }\left(x\right)=R_{\theta }\left(x\right)\left(\begin{array}{cc} \alpha & \\ & 1 \end{array}\right)R_{\theta }{\left(x\right)}^{T},$$

where the matrix $R_{\theta }\left(x\right)$ performs rotation of θ w.r.t. the reference direction (e.g., the maximum curvature) and α>0 is a parameter controlling the degree of anisotropy.

In the spectral domain, the anisotropic heat kernel is given by:

(22) $$h_{\alpha \theta t}\left(x,x^{\prime }\right)=\sum_{k\geq 0}e^{-t\lambda_{\alpha \theta ,k}}\phi_{\alpha \theta ,k}\left(x\right)\phi_{\alpha \theta ,k}\left(x^{\prime }\right),$$

where $\phi_{\alpha \theta ,k}\left(x\right)$ and $\lambda_{\alpha \theta ,k}$ are the eigenfunctions and eigenvalues of the anisotropic Laplacian $\Delta_{\alpha \theta }=-\mathrm{div}\left(A_{\alpha \theta }\left(x\right)\nabla \right)$. In [120], such kernels were used as the weighting functions for the construction of the patch operator:

(23) $$\left(D\left(x\right)f\right)\left(\theta ,t\right)=\int_{X}h_{\alpha \theta t}\left(x,x^{\prime }\right)f\left(x^{\prime }\right)dx^{\prime }.$$

Similar to the spectral analysis-based intrinsic CNN, heat diffusion CNN is composed of sequentially stacked layers, i.e., the output of the previous layer is used as the input to the subsequent layer, and the convolution operation is replaced by a layer tailored for heat diffusion.

### 5.4. A Unified Spatial-Domain Geometric Deep Learning Architecture for Human Shape Analysis

The authors in [132] proposed a unified geometric CNN generalizing the CNN to non-Euclidean domains. Instead of using fixed handcrafted weight functions, parametric kernels with learnable parameters were proposed. Particularly, a Gaussian kernel with learnable parameters was used:

(24) $$w_{j}\left(u\right)=\exp\left(-\frac{1}{2}{\left(u-\mu_{j}\right)}^{T}\sum_{j}^{-1}\left(u-\mu_{j}\right)\right)$$

where $\sum_{j}$ and $\mu_{j}$ are learnable d×d and a d×1 covariance matrix and mean vector. Various non-Euclidean CNN methods previously proposed in the literature can be considered as particular instances of the proposed framework.

### 5.5. Geometric Structures over Deep Learning for Human Action Recognition

There are also studies on learning geometric structures over CNN. For example, the authors in [133] proposed a deep discriminative structured model, namely convolutional neural random fields (CNRFs), and applied it to the action recognition problem. In the proposed model, a spatiotemporal CNN was developed for feature learning from input image frames, and the CNN was combined with conditional random fields (CRFs) for capturing the interdependencies between outputs. The parameters from both CRF and CNN were learned in a joint fashion, which enabled structured prediction and feature learning.

## 6. Generalized Geometrics for Human-Related Analysis

General information denoting spatial or temporal distributions and attribute occurrences can be considered as geometric information in a generalized perspective. They are also useful information for boosting HRA. They are named generalized geometrics and are further classified into three sub-categories and introduced in this section.

### 6.1. Spatial Geometrics for Human Pose-Related Analysis and Human Action-Related Analysis

In HPA, spatial geometrics can be encoded as local structural features. The authors in [134] proposed a local joint structure as a complement for global features of individual body part locations and combined the two features for posture description. Local joint structures, specifically the triangle area of the three consecutively adjacent joints, were computed. It is a complement for body part locations in the sense that body part locations denote a single body part, while the proposed joint structure contains relative joint positions. Then, classification was performed with a combined method of dynamic time warping, Fourier temporal pyramid representation, and linear SVM.

Some works directly explored the neighboring properties. For example, the authors in [135] proposed a geometric correspondence feature named the Trisarea feature. It describes neighboring properties between human body joints and is defined as the area of the triangle formed by three joints. This feature is utilized to identify human poses, of which variations over time capture the characteristics of human action.

Furthermore, in HAR, spatial geometrics can be encoded in features. For example, relative positions, distances between body joints, etc., are effective spatial geometric features. In [136], features from 3D skeleton data were processed separately by LSTM and CNN to conduct effective recognition with later fusion. Spatial features such as relative position, the distance between joints, and distances between joints and lines were explored, while temporal features such as the joint distances map and the joint trajectories map were studied. Spatial features were fed into LSTM, and temporal features were fed into CNN for recognizing actions.

Another way of encoding spatial geometrics is through modeling the co-occurrence of actions. The co-occurrence of actions was modeled in a probabilistic way without supervision in [137]. Videos containing human actions are considered as a sequence of short-term action clips (action words), and an activity is considered as a set of action topics indicating which actions are present in the video. A probabilistic model relating the action words and the action topics was proposed. It modeled long-range action relations that exist in the complex activity. The model was applied to unsupervised action segmentation and recognition and to detect forgotten actions, namely action patching.

### 6.2. Temporal Geometrics for Human Action Recognition

Temporal geometrics can be encoded by directly modeling the dynamics in a geometric space. For example, the authors in [138] proposed a second-order stochastic dynamical model in the state space (a Riemannian manifold) of articulated objects and derived equations of a Riemannian extended Kalman filter to perform the structure estimation from an image sequence captured by a camera from one perspective. The proposed model was proven by the authors to be locally weakly observable.

Furthermore, motion dynamics can be described in the original feature space. One widely-used measure is through scene flow. Scene flow describes the motion of 3D objects in the real world and implicitly describes the geometry of the 3D objects in motion. Scene flow can be considered as an optical flow fused from multiple cameras. The authors in [27] proposed the extraction and use of scene flow for action recognition from RGB-D data.

### 6.3. Spatial-Temporal Geometrics for Action Segmentation and Action Recognition

Action segmentation algorithms mine temporal segments containing actions from untrimmed videos. By incorporating a spatial component that represents the relationships between objects and a temporal component to capture object relationships across time, the method in [139] achieved better performances.

For action recognition problems, spatiotemporal information can be extracted through feature extraction and network extraction. The authors in [140] presented SkeletonNet, a deep learning framework for skeleton-based 3D action recognition. Cosine distance (CD) and normalized magnitude (NM) features were proposed and extracted from each frame of the skeleton sequence. Instead of treating the features of all frames as a time series, the authors fed extracted features to the proposed deep learning network, which contained two streams, one to extract the general features from the CD feature, while the other processed the NM feature. Outputs from the two streams were concatenated and processed by a fully-convolutional layer and then classified.

**Figure 18:**
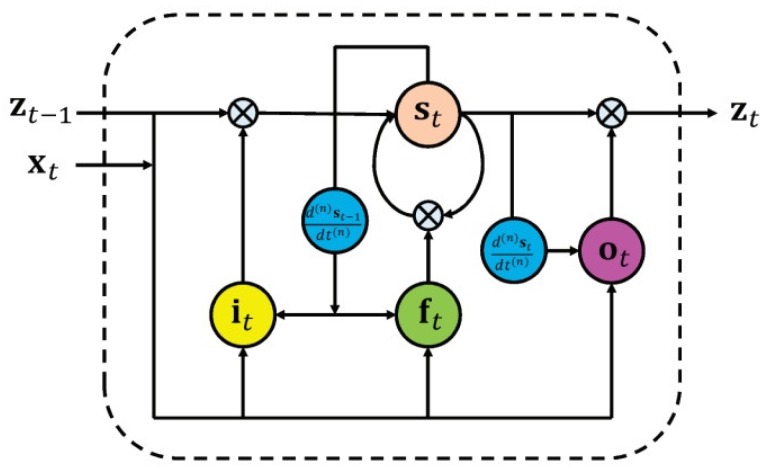
Architecture of the proposed dRNNmodel. In the memory cell, the input gate $i_{t}$ and the forget gate $f_{t}$ are controlled by the derivative of states (DoS) $\frac{d^{\left(n\right)}s_{t-1}}{dt^{\left(n\right)}}$ at t-1, and the output gate $o_{t}$ is controlled by the DoS $\frac{d^{\left(n\right)}s_{t}}{dt^{\left(n\right)}}$ at *t* [141]. ©2015 IEEE. Reprinted, with permission, from V. Veeriah, N. Zhuang, and G. Qi, Differential Recurrent Neural Networks for Action Recognition, *in Proceedings of the IEEE International Conference on Computer Vision*, Región Metropolitana, Chile, 11–18 December 2015, 4041–4049.

Furthermore, spatiotemporal information can be considered by modifying the deep learning network structure. For example, the authors in [141] proposed a differential gating scheme for a long short-term memory (LSTM) neural network and incorporated the spatial dynamics in action motions. The information gain was achieved by the derivative of states (DoS). The LSTM neural network utilizes three types of gating schemes for learning representations from long input sequences. The proposed method considered spatial information by incorporating DoS from the previous state into the input and forget gate and DoS from the current state into the output gate (as shown in Figure 18). Another example is [142], in which the authors extended the RNN-based methods from temporal domains to spatiotemporal domains and applied them to analyze action-related information within the input data.

The spatiotemporal information can also be learned from channels other than RGB data. The authors in [26] combined spatiotemporal geometric features from depth images and joint positions to solve human action recognition problems. The method learned spatiotemporal features by constructing a 3D-based deep CNN (3D${}^{2}$CNN) for depth sequences. Depth images and joint positions were processed separately and fused in a later stage. Furthermore, spatiotemporal discrimination can be utilized to recognize human actions at different speeds. For example, the authors in [143] achieved this through considering spatiotemporal discrimination and action speed variations.

## 7. Validation Datasets

Publicly-available datasets for validating HRA are collected and categorized according to data types and applications. The datasets are classified based on their data type and their targeted applications: 3D human datasets, composed of 3D human models mainly for human shape analysis; 3D human action datasets with 3D data for human action analysis; RGB-D people datasets, composed of RGB-D data for people detection and people tracking; RGB-D human pose datasets for human pose analysis; and RGB-D human action datasets with RGB-D data for human action analysis.

### 7.1. 3D Human Datasets

In this section, public datasets on 3D humans are collected. These datasets are utilized to validate applications such as shape analysis, including deformable shape matching and shape retrieval. 3D human data with noise and partial 3D human model analysis are also considered.

#### 7.1.1. KIDS Dataset

**Figure 19:**
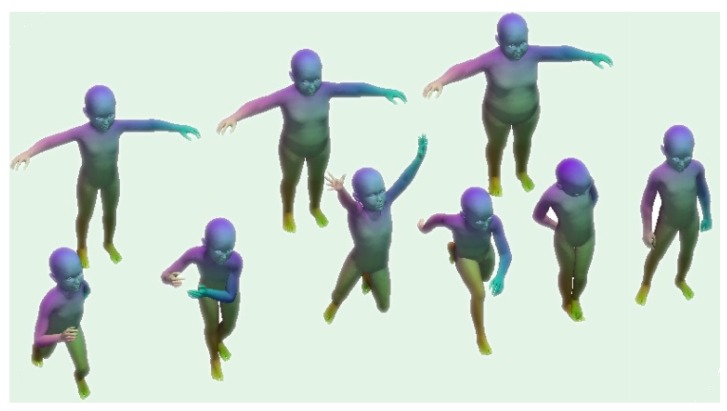
Examples from the Kidsdataset. The figure is originally from [94].

This dataset (https://vision.in.tum.de/data/datasets/kids) consists of two shape classes (“kid” and “fat kid”, as shown in Figure 19) under different poses, where the same poses are applied to both classes. The 3D shapes undergo nearly isometric and within-class deformations. All shapes in the dataset are given in OFF format and have around 60k vertices and consistent triangulations.

#### 7.1.2. ShapeNet

ShapeNet is a well-maintained, large-scale dataset of 3D shapes. ShapeNet is composed of several subsets:

(1) ShapeNetCore [144], including 55 common object categories (approximately 51,300 unique 3D models), 12 object categories of PASCAL 3D+, and a popular computer vision 3D benchmark dataset.
(2) ShapeNetSem [145], including 12,000 models of 270 categories and annotated with manually-verified category labels, consistent alignments, real-world dimensions, estimates of their material composition at the category level, and estimates of their total volume and weight.

In ShapeNet, there are 35 subcategories (such as “adult, grownup”, “worker”, “child, baby”, etc.) and 2561 human-related models, namely “person, individual, someone, somebody, mortal, soul” in the “natural object” category.

#### 7.1.3. TOSCA High-Resolution Dataset

The TOSCA [146] dataset (http://tosca.cs.technion.ac.il/book/resourcesunderlinetag_data.html) includes high-resolution 3D nonrigid shapes in variant poses. The dataset contains 80 object categories, including 11 cats, 9 dogs, 3 wolves, 8 horses, 6 centaurs, 4 gorillas, 12 females, and 2 males. Typically, the model has approximately 50,000 vertices.

#### 7.1.4. Human 3.6M

This dataset (http://vision.imar.ro/human3.6m/description.php) is composed of 3.6 million 3D human poses with corresponding images. The dataset contains 11 professional actors, including 6 males and 5 females, and 17 scenarios, including “discussion”, “smoking”, “taking a photo”, “talking on the phone”, etc.

The dataset is composed of high-resolution 50-Hz videos from four calibrated cameras. The dataset has rich annotations, including accurate 3D joint positions, joint angles from a high-speed motion capture system, where 24 pixel-level body part labels for each configuration are given. It also provides accurate background subtraction and person bounding boxes.

Furthermore, the Human 3.6M dataset provides precomputed image descriptors, software for visualization and discriminative human pose prediction, and performance evaluation on a withheld test set [147,148].

#### 7.1.5. H3D Database

**Figure 20:**
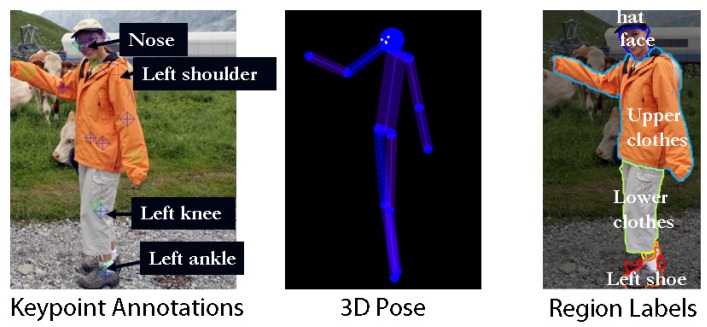
Contents from the H3D dataset. The figure is originally from the website [149].

H3D (Humans in 3D) (https://www2.eecs.berkeley.edu/Research/Projects/CS/vision/shape/h3d/) is a 3D human dataset with annotations. The annotations include the joints, other keypoints (“eyes”, “ears”, “nose”, “shoulders”, “elbows”, “wrists”, “hips”, “knees”, and “ankles”), and 3D poses inferred from the keypoints with a visibility Boolean for each keypoint. The dataset is also annotated with regions (“upper clothes”, “lower clothes”, “dress”, “socks”, “shoes”, “hands”, “gloves”, “neck”, “face”, “hair”, “hat”, “sunglasses”, “bag”, “occluder”), and body types (“male”, “female”, or “child”). For detailed descriptions, please refer to [149]. Figure 20 shows an example of the data and their annotations.

#### 7.1.6. 3D Shape Dataset with Noise

This dataset (https://vision.in.tum.de/data/datasets/topkids) consists of a collection of 3D shapes under deformations including topological changes [20]. The dataset has the ground-truth matching the null shape for all shapes, but not all vertices have a match due to topological changes.

#### 7.1.7. Partial Shape Dataset

**Figure 21:**
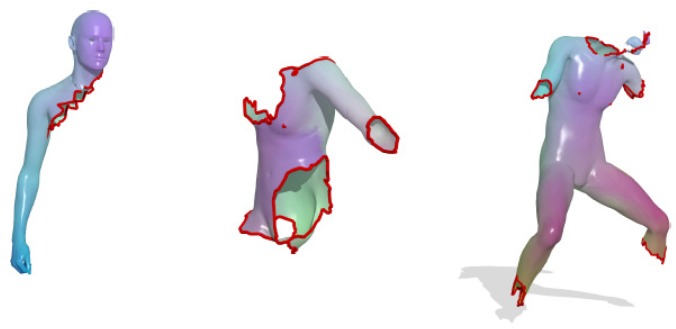
Examples from the Partial Shape Dataset. The figure is originally from the website [18].

The Partial Shape Dataset (https://vision.in.tum.de/data/datasets/partial) includes two datasets, one is the cuts dataset with 456 partial shapes and the other is the holes dataset with 684 partial shapes. These two datasets exemplify different kinds of partiality: The cuts dataset contains shapes with a single cut; The holes dataset contains irregular holes and multiple cuts. Examples from the dataset are shown in Figure 21. The datasets provided can be used for deformable 3D shape matching and retrieval under partiality transformations [18].

#### 7.1.8. SHREC

The 3D Shape Retrieval Contest (http://www.shrec.net) evaluates the effectiveness of 3D shape retrieval algorithms. SHREC’18 is the tenth edition of the contest (https://3dor2018.sites.uu.nl). The contest contains tracks with various goals. Many tracks are related to the scope of this paper; for example, shape retrieval from 3D human shapes represented by triangular meshes (https://vision.in.tum.de/~laehner/shrec2016/), human shape retrieval from depth sensor data (http://www.andreagiachetti.it/shrec16/), and partial shape matching (http://tosca.cs.technion.ac.il/book/resourcesunderlinetag|data.html) based on the TOSCA high-resolution dataset [146] (http://tosca.cs.technion.ac.il/book/resourcesunderlinetag|data.html).

### 7.2. 3D Human Action Datasets

#### 7.2.1. CMU Graphics Lab Motion Capture Database

The dataset (http://mocap.cs.cmu.edu/) contains 2605 motion capture trials of six categories, including “human interaction”, “interaction with environment”, “locomotion”, “physical activities & sports”, “situations & scenarios”, and “test motions”, and 23 subcategories, including “running”, “walking”, “jumping“, etc.

#### 7.2.2. HumanEva Dataset

The HumanEva-I and HumanEva-II (http://humaneva.is.tue.mpg.de/) datasets were obtained from a motion capture system. The HumanEva-I dataset contains seven calibrated video sequences (four grayscale and three color) with synchronized 3D body poses. The dataset has 4 subjects of 6 actions, including “walking”, “jogging”, “gesturing”, etc. The dataset is split into training, validation, and testing sets. Also, the error measurements of the 2D and 3D poses are provided.

### 7.3. RGB-D People Datasets

#### 7.3.1. RGB-D People Datasets

**Figure 22:**
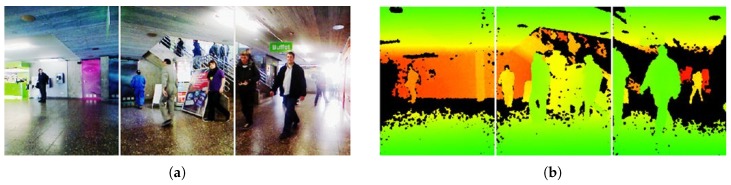
Examples from the RGB-D People Dataset. The figure shows the color image data (**a**) and the dense depth data (**b**) of three examplar frames. The figure is originally from the website [150,151].

The RGB-D People Datasets (http://www2.informatik.uni-freiburg.de/~spinello/RGBD-dataset.html) contain people in RGB-D Kinect data with annotaions. This datasets are composed of more than 3000 RGB-D frames. In the datasets, mostly are upright walking and standing persons. The persons are under differnt occlusion conditions. This dataset has been re-annotated in [150,151]. Examples from the dataset are shown in Figure 22.

#### 7.3.2. RGB-D Human Tracking Dataset

**Figure 23:**
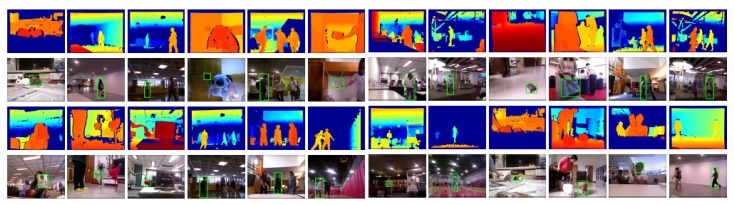
Contents from the RGB-D Human Tracking Dataset. The figure is originally from the RGB-D Human Tracking Dataset website [152].

There are five validation videos with ground-truths, and 95 evaluation videos in the RGB-D Human Tracking Dataset (http://tracking.cs.princeton.edu/dataset.html). Captured by Kinect v1, each sequence has its RGB images and depth images. Captured videos contain moving objects such as humans, balls, and cars and are labeled with per-frame bounding boxes covering only the target object. The authors in [152] presented a quantitative comparison of various algorithms on this dataset. Examples and annotations from the dataset are shown in Figure 23.

### 7.4. RGB-D Human Pose and Posture Datasets

#### Kinect Gesture Dataset

The Microsoft Research Cambridge-12 Kinect gesture dataset (https://www.microsoft.com/en-us/download/details.aspx?id=52283ampersandtag|from\=http%3A%2F%2Fresearch.microsoft.com%2Fen-us%2Fum%2Fcambridge\%2Fprojects%2Fmsrc12%2F) is composed of sequences of human movements. Human gestures are denoted by body part locations. The dataset contains 594 sequences and 719,359 frames performed by 30 people with 12 gestures. There are 6244 gesture instances in total in the dataset.

### 7.5. RGB-D Human Action and Activity Datasets

#### 7.5.1. Human Daily Activity Dataset

The authors in [137] collected an RGB-D activity video dataset recorded by the Kinect v2, containing human daily activities composed of multiple actions interacting with different objects.

#### 7.5.2. Cornell Activity Datasets

Cornell Activity Datasets CAD-60 and CAD-120 are two RGB-D human activity datasets (http://pr.cs.cornell.edu/humanactivities/data.php) containing skeleton and RGB-D data. RGB-D data have a resolution of 240 × 320, of which the RGB data are saved as three-channel 8-bit PNG files, and the depth data are saved as single-channel 16-bit PNG files.

The CAD-60 dataset contains 60 RGB-D videos, performed by 4 subjects, including 2 males, 2 females, and 1 left-handed person, in 5 different environments, including “office”, “kitchen”, “bedroom”, “bathroom”, and “living room”, and of 12 activities, including “rinsing mouth”, “brushing teeth”, “wearing contact lens”, etc.

The CAD-120 dataset contains 120 RGB-D videos of long daily activities, 4 subjects (same as CAD-60), 10 high-level activities (“making cereal”, “taking medicine”, “stacking objects”, etc.), 10 sub-activity labels (“reaching”, “moving”, “pouring”, etc.), and 12 object affordance labels (“reachable”, “movable”, “pourable”, etc.).

#### 7.5.3. 50 Salads Dataset

The dataset (http://cvip.computing.dundee.ac.uk/datasets/foodpreparation/50salads/) captures 25 people preparing two mixed salads each and contains over four hours of the annotated accelerometer and RGB-D video data. The RGB video data have a resolution of 640 × 480 pixels at 30 Hz and the depth maps a resolution of 640 × 480 pixels at 30 Hz, and the three-axis accelerometer data are at 50 Hz [153].

#### 7.5.4. UR Fall Detection Dataset

**Figure 24:**
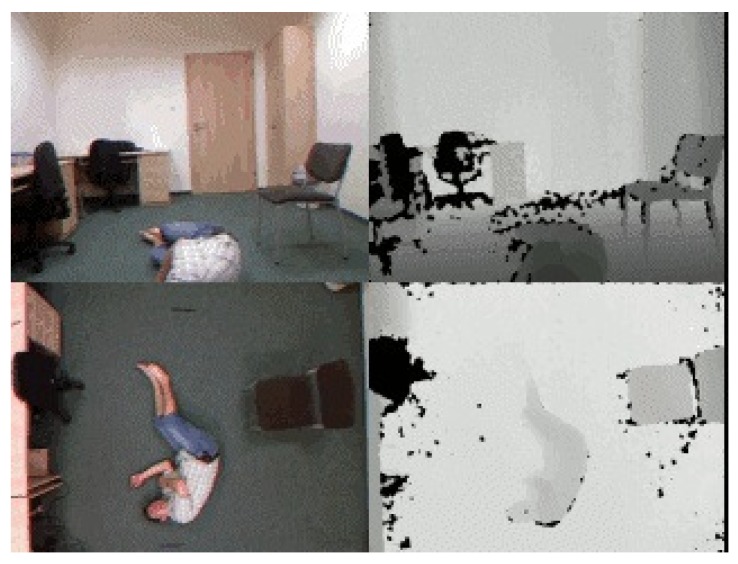
Sample Images from the URfall dataset. The figure was captured from the demo video on the UR Fall Dataset website [154].

This dataset (http://fenix.univ.rzeszow.pl/~mkepski/ds/uf.html) contains 70 (30 falls + 40 activities of daily living) sequences [154]. Fall events were recorded with two Microsoft Kinect cameras and corresponding accelerometric data. Examples from the dataset are shown in Figure 24.

#### 7.5.5. Tum Kitchen Dataset

The TUM Kitchen Dataset contains several subjects sitting by a table. Some perform activities simulating a robot, transporting items one-by-one; while others behave more human-like and grasp as many objects as they can in one performance. And for each subject performing reaching and grasping, there are two trials.

## 8. Performances of Related Works

The performances of various geometric methods for HRA studied in this review are compared in terms of estimation accuracy or estimation error and shown in Table 1. The methods are categorized based on their applications, i.e., HSA, HPA, or HAA. Due to the characteristics of the specific application, the number of methods in each category varies. For example, for HRA and HPA, many algorithms measure their performance by quality, while for HAA, many algorithms validate their performance based on recognition accuracy. In each category, the methods are listed in the chronological order of publication, and then in alphabetical order by the method names. For each validation dataset, the best recognition accuracy (in percent) or the minimum estimation error (in centimeter) among all experiment settings are listed.

From the table, we can see that the average precision of HRA and mostly HAA is quite high. Except for some difficult datasets (i.e., “PASCAL VOC 2011”, “PASCAL VOC 2012”, “ChaLearn LAP IsoGD”, “50 Salads”, and “JIGSAWS”), the recognition accuracy was above 80% for all validation datasets. The “Enhanced-LSTM-based method” and “Gram matrix-based method” achieved 100% accuracy on three of the validation datasets.

**Figure 25:**
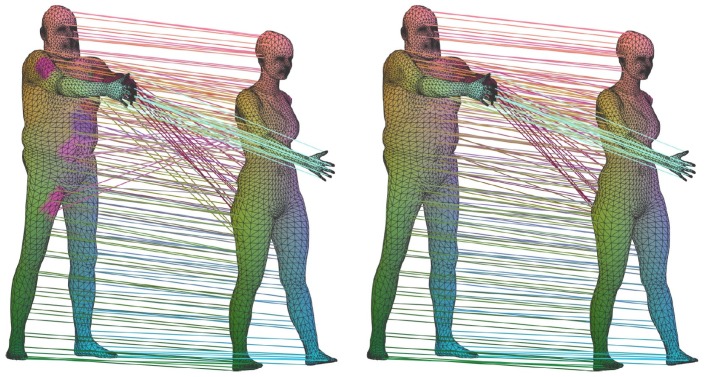
The figure shows exemplary point-to-point maps from one human body model to another. The overall performance of the proposed geometric method (right) is working better than the compared SHOT(left) method on the entire shape [83]. Republished with permission of ACM, from ACM Trans. Graph., E. Corman and M. Ovsjanikov, Vol. 38, 2019; permission conveyed through Copyright Clearance Center, Inc.

For HSA, the paper reviews the related works on human shape correspondence, human model symmetry analysis, and human shape recognition. For human shape correspondence, the authors in [83] represented a deformation field as a linear operator on real-valued functions on the shape and gave the state-of-the-art performance on human shape correspondence. An exemplary result is illustrated in Figure 25. Another exemplary work is from [95], and its visualized results are illustrated in Figure 12. Quantitative measurement of the method from [126] is shown in the human shape analysis section in Table 1.

**Figure 26:**
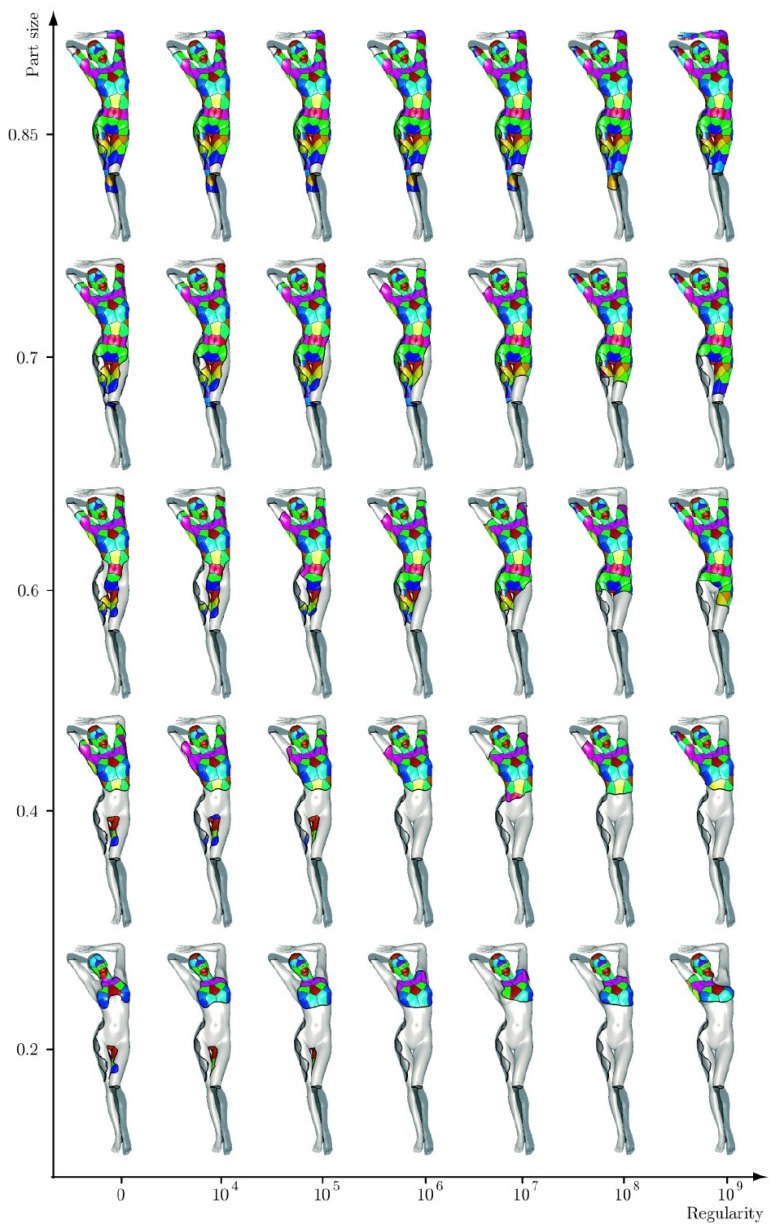
The figure shows exemplary results on partial symmetries of human body models. The partial human body models are obtained by removing certain body parts, and the removed body parts are marked in semitransparent dark gray. The experiments are carried out under various regularization coefficients (the horizontal axis) and various body part sizes (the vertical axis). Symmetric body parts are marked with the same color. Discarded body parts are marked in light gray [84]. Reprinted by permission from SPRINGER NATURE: Springer Nature, Int. J. Comput. Vis., Full and Partial Symmetries of Non-rigid Shapes, D. Raviv, A. M. Bronstein, M. M. Bronstein, R. Kimmel, Copyright 2010.

For human model symmetry analysis, the authors in [84] proposed a numerical framework for the analysis, addressing the problems of full and partial exact and approximate symmetry detection and classification. The exemplary results are illustrated in Figure 26. Note that the increase in regularity results in the shortening of the boundary at the expense of the symmetry of the part.

**Figure 27:**
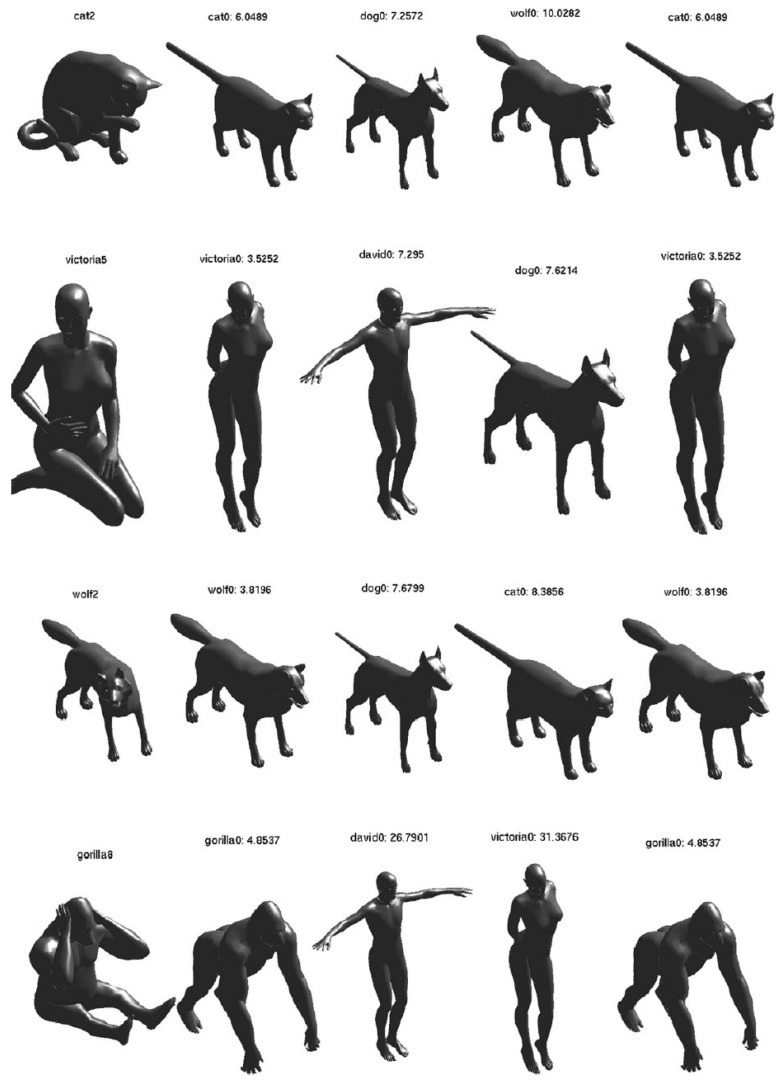
The figure shows exemplary shape recognition results. The first column denotes the query shape, and the second to the fourth columns show the three closest matches [86]. Reprinted from Pattern Recognition, Vol. 45, D. Smeets, J. Hermans, D. Vandermeulen, P. Suetens, Isometric Deformation Invariant 3D Shape Recognition, 2817–2831, Copyright 2012, with permission from Elsevier.

For human body shape recognition, a state-of-the art method [86] was proposed based on a geodesic distance matrix. A recognition rate of 100% was obtained on the TOSCA database. Some exemplary results are illustrated in Figure 27.

**Figure 28:**
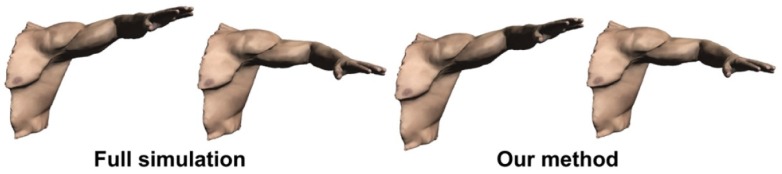
The proposed method produces a good approximation to the full simulation while being 60-times faster. The figure is originally from [102]. Reproduced with permission from Jernej Barbic, ACM Transactions on Graphics; published by ACM Digital Library, 2016.

For HPA, the review studies human pose space modeling and human pose estimation. The method of using a pose-space subspace method [102] gives a good performance on modeling the human pose space. The proposed method uses secondary soft-tissue finite element method (FEM) dynamics computed under arbitrary rigged or skeletal motion. Experiment comparisons are illustrated in Figure 28. The performances of the human pose estimation method using geometric methods are shown in Table 1.

## 9. Conclusions and Discussions

This review presented a comprehensive study on human-related analysis (HRA), including human shape analysis, human pose-related analysis, and human action-related analysis. It first introduced fundamental concepts in the topology and manifold as fundamental knowledge for geometric modeling with these theories. Then, geometric methods using these theories were introduced. Later, geometric methods applied for HRA were studied. Considering the great impactof deep learning and its potential in feature extraction and feature representation, the review also considered geometric deep learning, which has recently been a popular topic. Then, generalized geometric methods, which study general purpose geometric information for HRA, were explored. Validation datasets for verifying geometric HRA methods were collected, and the performances of various methods were collected, compared, and shown in a table.

For further research, one topic worth exploring is defining intrinsic deep learning algorithms on RGB-D data, specifically defining the convolution, the pooling, and the spatial shift operation on the RGB-D domain. There are very few works on this topic despite its wide applications. Another research topic worth exploring is learning geometric information and utilizing it as priors. 3D data are still comparatively more difficult to acquire than images or videos; thus, it would be helpful to utilize geometric information as priors for improving task performances, which use images or videos as inputs.

## Figures and Tables

**Table 1:** Evaluation comparisons of geometric methods.

Applications	Year	Methods	Validation Datasets	Accuracy (%) or Error (cm)
Human Shape Analysis	2016	Dense correspondence-based method [126]	FAUST	2–2.35 cm
			CMUMocap	89.46%
Human Pose Related	2017	SkeletonNet [140]	NTURGB+D	81.16%
Analysis			SBUKinect interaction	93.47%
	2011	Spatio temporal manifold model-based method [111]	Mocap	90.00%
	2012	Bi-lingual Hankelets [45]	IXMAS	90.57%
	2012	Graph matching-based method [105]	KTH	89.3%
	2013	Directed acyclic graph kernel-based method [107]	UCFSport	85.2%
	2014	Fully-convolutional network-based method [113]	PASCAL VOC 2011	62.7%
			MSRAction3D	92.46%
	2014	Lie group-based method [109]	UTKinect-Action	97.08%
			Florence3D-Action	90.88%
	2014	Shape matching-based method [94]	TOSCA	90.00%
	2015	Deep deconvolution network-based method [114]	PASCAL VOC 2012	72.5%
			KTH-1	93.96%
	2015	Differential recurrent neural network-based method	KTH-2	92.12%
		[141]	MSR Action3D	92.03%
	2016	3D DCNN-based method [26]	MSR Action3D	98.14%
			Weizmann	98.88%
	2016	Convolutional neural random fields [133]	Youtube	94.4%
			UCF50	86.5%
			WBJR	95.70%
			NTURGB+D	81.60%
			SBUInteraction	93.3%
	2016	Enhanced-LSTM-based method [142]	UT-Kinect	95.00%
			Berkeley MHAD	100.00%
			MSRAction3D	94.80%
			HDM05	88.0±6.3%
Human action related	2016	Gram matrix-based method [104]	MSR-Action3D	96.97%
Analysis			MHAD	100%
			UTKinect	100%
			MSR Action3D	85.86%
	2016	Local joint structure and body part locations	UTKinect-Action	96.49%
		Feature-based method [134]	Florence3D-Action	87.47%
			MSR Action3D	94.77%
	2016	Motionlet-graph-based method [108]	Florence 3D Actions	91.63%
			UTKinect Action	97.44%
			Florence3D	92.16%
			G3D	92.12%
	2016	Relative 3D geometry-based method [103]	MSR Action3D	90.69%
			MSRPairs	94.33%
			UTKinect-Action	97.20%
			Florence3D	91.40%
	2016	Rolling map-based method [110]	MSRPairs	94.67%
			G3D	90.94%
	2016	Segmental spatiotemporal CNN-based method [139]	50 Salads	72.00%
			JIGSAWS	74.22%
	2017	LSTM and CNN-based method [136]	NTU RGB+D	87.40%
	2017	Geometric feature pooling-based method [136]	HOIactivity dataset	89.6%
	2017	Scene flow to action map [27]	ChaLearn LAP IsoGD	36.27%
			Multi-modal and multi-view and interactive dataset	91.2%
	2017	Spatiotemporal feature-based method [143]	MSRAction3D	93.81%
			UTKinect-Action	97.47%

**Figure A1:**
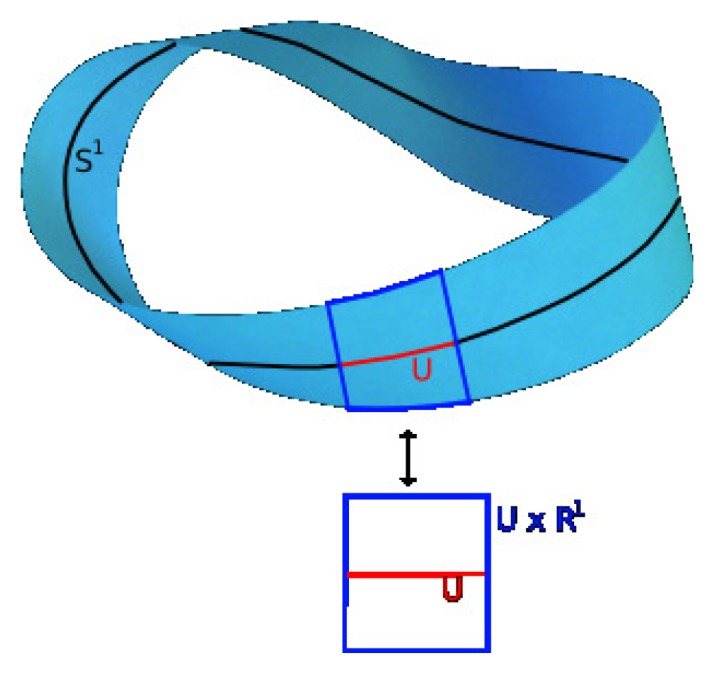
Vector bundle illustration (by Jakob.scholbach at English Wikipedia, CC BY-SA 3.0, https://commons.wikimedia.org/w/index.php?curid=6082417).

## Abbreviations

**Set Theory Symbols**	
∼	A relation
[x]	The equivalence class *x*
*V*/${∼}_{W}$	The quotient space of *V* by *W*, also denoted as *V*/*W*
**Topological Symbols**	
Ω	A topological space
*G*	A topological group
GL	The general linear group
**Manifold Symbols**	
M	A manifold
$T_{x}M$	The tangent space of M at *x*
TM	The tangent bundle of M
$T^{*}M$	The cotangent bundle
Γ(F)	The section of the vector bundle F
Exp	The exponential map
Log	The inverse exponential map, also denoted as Exp${}^{-1}$
∇	A connection
$\nabla^{*}$	A dual connection
γ	A geodesic, i.e., a curve γ:I→M such that $\nabla_{\gamma \prime }\gamma \prime =0$.
$\rho_{g}$	M×M→R, the geodesic distance function determined by *g*,
(M,g)	A Riemannian manifold equipped with a metric *g*
(M,M)	A smooth manifold of a pair of a topological manifolds M and an atlas M on M

## Appendix A. Mathematical Concepts

Expanded geometric concepts are introduced in the Appendix. The concepts are categorized following the mathematical branch to which they belong. Appendix A.1 introduces expanded concepts from the set theory. Appendix A.2 explains expanded concepts from topology. Appendix A.3 introduces expanded concepts from algebraic topology, and Appendix A.4 introduces extended manifold concepts.

### Appendix A.1. Set Theory Concepts

#### Appendix A.1.1. Equivalence Relations

A (binary) relation on a set *X* is a subset of X×X. We often denote relations by ∼ and write $x∼x^{\prime }$ to indicate that $\left(x,x^{\prime }\right)$ is in the relation.

A relation ∼ on a set *X* is an equivalence relation if the following three conditions hold:

- x∼x for all x∈X.
- $x∼x^{\prime }$ if and only if $x^{\prime }∼x$.
- $x∼x^{\prime }$ and $x^{\prime }∼x^{\prime \prime }$ implies $x∼x^{\prime \prime }$.

#### Appendix A.1.2. Equivalence Class

The ways to denote equivalence classes are listed as follows:

- The equivalence class of x∈X, denoted by [x], means the set $\left\{x^{\prime }|x∼x^{\prime }\right\}$. The sets [x] for all x∈X form a partition of the set *X*.
- The set of equivalence classes under ∼ can be denoted as X/∼, and it is referred to as the quotient of *X* with respect to ∼.

#### Appendix A.1.3. Covering

A family $A_{i},i\in I$ of subsets of *X* is a covering of *X* if $X={⋃}_{i\in I}A_{i}$.

### Appendix A.2. Topological Concepts

#### Appendix A.2.1. Closed Sets/Interior and Closure of A Set/Limit Points

A subset *A* of a topological space *X* is said to be closed if the set X-A is open.

Given a subset *A* of a topological space *X*, the interior of *A* is defined as the union of all open sets contained in *A*, and the closure of *A* is defined as the intersection of all closed sets containing *A*.

If *A* is a subset of the topological space *X* and if *x* is a point of *X*, we say that *x* is a limit point (or “cluster point,” or “point of accumulation”) of *A* if every neighborhood of *x* intersects *A* at some point other than *x* or *x* is a limit point of *A* if it belongs to the closure of A-[x]. The point *x* may or may not lie in *A*.

#### Appendix A.2.2. Continuous Function

Let *X* and *Y* be topological spaces. A function f:X→Y is continuous if for each point *x* of *X* and each neighborhood *N* of f(x) in *Y*, the set $f^{-1}\left(N\right)$ is a neighborhood of *x* in *X*.

Formulated with the concept of openness, a function from *X* to *Y* is continuous if and only if the inverse image of each open set of *Y* is open in *X*.

#### Appendix A.2.3. Quotient Map

Let *X* and *Y* be topological spaces and p:X→Y be a surjective map. The map *p* is said to be a quotient map provided a subset *U* of *Y* that is open in *Y* if and only if $p^{-1}\left(U\right)$ is open in *X*.

#### Appendix A.2.4. Metric

A metric on a set *X* is a function d:X×X→R having the following properties:

- d(x,y)≥0 for all x,y∈X, equality holds if and only if x=y.
- d(x,y)=d(y,x) for all x,y∈X.
- (Triangle inequality) d(x,y)+d(y,z)≤d(x,z), for all x,y,z∈X.

A topology can be imposed on a set by defining a metric on a topology. Given a metric *d* on *X*, d(x,y) is often called the distance between *x* and *y*. Given t>0, consider the set $B_{d}\left(x,\epsilon \right)=\left\{y|d\left(x,y\right)<\epsilon \right\}$ of all points *y* whose distance from *x* is less than ϵ. It is called the ϵ-ball centered at *x*.

#### Appendix A.2.5. Hausdorff Space

For every pair of points p,q∈M in a Hausdorff space, there are disjoint open subsets U,V⊂M such that p∈U and q∈V.

### Appendix A.3. Algebraic Topology Concepts

#### Appendix A.3.1. Orbit Space

Let *X* be a space and *G* be a topological group; an action of *G* on *X* is a continuous map a:G×X→X such that (denoting a(g×x) by g·x):

- e·x=x for all x∈X.
- $g_{1}\cdot \left(g_{2}\cdot x\right)=\left(g_{1}\cdot g_{2}\right)\cdot x$ for all x∈X and $g_{1},g_{2}\;\in G$.

Define x∼g·x for all *x* and *g*, and the resulting quotient space is denoted X/G and called the orbit space of the action *a*.

#### Appendix A.3.2. Homotopy

A homotopy between two maps f:X→Y and g:X→Y is a map H:X×I→Y such that H(x,0)=f(x) and H(x,1)=g(x) for all x∈X. We then say that *f* and *g* are homotopic and write f≃g, or H:f≃g or $f\overset{H}{≃}g$.

A special case is when *f* is a path in *X*. If f:[0,1]→X is a continuous map such that $f\left(0\right)=x_{0}$ and $f\left(1\right)=x_{1}$, we say that *f* is a path in *X* from $x_{0}$ to $x_{1}$. We also say that $x_{0}$ is the initial point, and $x_{1}$ the final point of the path *f*.

#### Appendix A.3.3. Fundamental Group

Let *X* be a space and $x_{0}$ be a point of *X*. A path in *X* that begins and ends at $x_{0}$ is called a loop based at $x_{0}$. The set of path homotopy classes of loops based at $x_{0}$ with the operation * is called the fundamental group of *X* relative to the base point $x_{0}$. It is denoted by $\pi_{1}\left(X,x_{0}\right)$. Two spaces that are homeomorphic have fundamental groups that are isomorphic.

#### Appendix A.3.4. Homology

Although homotopy is easy to define and conceptually very attractive, homotopy groups are very difficult to compute. Another invariant is homology, which, instead of being easy to define and difficult to compute, is difficult to define and easy to compute. The idea of counting occurrences of patterns directly is unworkable, but that of counting equivalence classes of occurrences of patterns under an equivalence relation is workable. In this section, basic notations for homology, i.e., the definition of homology, the normal subgroup, the Abelian Group, and the commutator subgroup, are introduced.

##### Appendix A.3.4.1. Normal Subgroup

Let *G* be a group and *H* be a subset of *G*. If *H* is a group under the operation of *G*, then we say that *H* is a subgroup of *G*.

A subgroup *H* of a group *G* is normal in *G* if and only if gH=Hg for all *g* in *G*.

##### Appendix A.3.4.2. Abelian Group

A group is Abelian if xy=yx for all group elements *x* and *y*.

##### Appendix A.3.4.3. Commutator Subgroup

Let *G* be a group. If x,y∈G, we denote the element $\left[x,y\right]=xyx^{-1}y^{-1}$ by [x,y]; it is called the commutator of *x* and *y*. The subgroup of *G* generated by the set of all commutators in *G* is called the commutator subgroup of *G* and is denoted by [G,G]. The subgroup [G,G] is a normal subgroup of *G*, and the quotient group G/[G,G] is Abelian.

##### Appendix A.3.4.4. Homology

If *X* is a path-connected space, let $H_{1}\left(X\right)=\pi_{1}\left(X,x_{0}\right)/\left[\pi_{1}\left(X,x_{0}\right),\pi_{1}\left(X,x_{0}\right)\right]$. $H_{1}\left(X\right)$ is the first homology group of *X*. Groups $H_{n}\left(X\right)$ are called the homology groups of *X* that are defined for all n≥0.

### Appendix A.4. Manifold Concepts

#### Appendix A.4.1. Atlas

A family ${\left(U_{\alpha },u_{\alpha }\right)}_{\alpha \in A}$ of charts on M such that the $U_{\alpha }$ form a cover of M is called an atlas. An atlas A is called a smooth atlas if any two charts in A are smoothly compatible with each other.

#### Appendix A.4.2. Smooth Manifold

A smooth structure on a topological *n*-manifold M is a maximal smooth atlas. A smooth manifold is a pair (M,A), where M is a topological manifold, and A is a smooth structure on M. The smooth structure is usually omitted, and M is called a smooth manifold. Smooth structures are also called differentiable structures or $C^{\infty }$ structures. Note that a $C^{0}$ manifold is a topological manifold.

#### Appendix A.4.3. Section

If $\pi :\tilde{M}\to M$ is any continuous map, a section of π is a continuous map $\sigma :M\to \tilde{M}$ such that $\pi \circ \sigma =Id_{M}$. A local section is a continuous map σ:U→M defined on some open set U⊂M and satisfying the analogous relation $\pi \circ \sigma =Id_{U}$.

#### Appendix A.4.4. Vector Bundle/Fiber Bundle

A mapping f:E→M between manifolds is called a submersion at x∈E if the mapping of $T_{x}f:T_{x}E\to T_{f(x)}M$ is surjective or the rank of the mapping equals dimM.

A triple (E,p,M) is called a fibered manifold if p:E→M is a surjective submersion.

Let p:E→M be a smooth mapping between manifolds; a vector bundle chart on (E,p,M) means a pair (U,ψ), where *U* is an open subset in M and ψ is a fiber-respecting diffeomorphism as in the following diagram:  Here, *S* is a fixed finite-dimensional real (unless otherwise specified) vector space called the standard fiber or the typical fiber, *p* is a surjective submersion, and $\psi :p^{-1}\left(U\right)\to U\times S$ such that ${\mathrm{pr}}_{1}\circ \psi =p$.

Two vector bundle charts $\left(U_{1},\psi_{1}\right)$ and $\left(U_{2},\psi_{2}\right)$ are called compatible, if $\psi_{1}\circ \psi_{2}^{-1}$ is a fiber linear isomorphism, i.e., $\left(\psi_{1}\circ \psi_{2}^{-1}\right)\left(x,v\right)=\left(x,\psi_{1,2}\left(x\right)v\right)$ for some mapping $\psi_{1,2}:U_{1,2}U_{1}\cap U_{2}\to GL\left(V\right)$.

A vector bundle atlas ${\left(U_{\alpha },\psi_{\alpha }\right)}_{\alpha \in A}$ for (E,p,M) is a set of pairwise compatible vector bundle charts $\left(U_{\alpha },\psi_{\alpha }\right)$ such that ${\left(U_{\alpha }\right)}_{\alpha \in A}$ is an open cover of M.

A vector bundle (E,p,M) consists of a manifold E (the total space), a manifold M (the base), and a smooth mapping p:E→M (the projection) together with an equivalence class of vector bundle atlases. Figure A1 shows an exemplary vector bundle, where a Möebius strip is a line bundle over the one-sphere.

A fiber bundle (E,p,M,S) consists of a manifold E, a manifold M, a vector space *S*, and a smooth mapping *p* satisfying the above conditions.

#### Appendix A.4.5. The Tangent Bundle Of A Vector Bundle

Let (E,p,M) be a vector bundle with fiber addition ${+}_{E}:E\times_{M}E\to E$ and fiber scalar multiplication $m_{t}^{E}:E\to E$. Then, the tangent bundle of the manifold E, denoted by $\left(TE,\pi_{E},E\right)$, is itself a vector bundle with fiber addition denoted by ${+}_{TE}$ and scalar multiplication denoted by $m_{t}^{TE}$.

#### Appendix A.4.6. Vertical Bundle

Given two fibered manifolds (M,p,N) and $\left(\bar{M},\bar{p},\bar{N}\right)$, a morphism $\left(M,p,N\right)\to \left(\bar{M},\bar{p},\bar{N}\right)$ means a smooth map f:M→N transforming each fiber of M into a fiber of $\bar{M}$.

Two maps f,g:M→N are said to determine the same *r*-jet (jets or holonomic jets) at x∈M, if for every curve γ:R→M with γ(0)=x, the curves f∘γ and g∘γ have the $r^{\mathrm{th}}$ order contact at zero. In such a case, we write $j_{x}^{r}f=j_{x}^{r}g$ or $j^{r}f\left(x\right)=j^{r}g\left(x\right)$.

The elements of the manifold $T_{k}^{r}MJ_{0}^{r}\left(R^{k},M\right)$ are said to be the *k*-dimensional velocities of order *r* on M, or (*k*,*r*)-velocities.

The projection $\pi_{r-1}^{r}:T^{r*}M\to T^{r-1*}M$ is a linear morphism of vector bundles. Its kernel is described by the following exact sequence of vector bundles over M:

$$0\to S^{r}T^{*}M\to T^{r*}M\overset{\pi_{r-1}^{r}}{\to }T^{r-1*}M\to 0$$

Let (E,p,M,S) be a fiber bundle and $T_{p}:TE\to TM$ be the tangent mapping. Its kernel ker$T_{p}=:VE$ is called the vertical bundle of E. For example, if ${\left(U_{\alpha },\psi_{\alpha }:E|U_{\alpha }\to U_{\alpha }\times V\right)}_{\alpha \in A}$ is a vector bundle atlas for E, such that $\left(U_{\alpha },u_{\alpha }\right)$ is also a manifold atlas for M, then ${\left(E|U_{\alpha },\psi_{\alpha }^{\prime }\right)}_{\alpha }\in A$ is an atlas for the manifold E, where:

$$\psi_{\alpha }^{\prime }:=\left(u_{\alpha }\times {\mathrm{Id}}_{V}\right)\circ \psi_{\alpha }:E|U_{\alpha }\to U_{\alpha }\times V\to u_{\alpha }\left(U_{\alpha }\right)\times V\subset R^{m}\times V.$$

Hence, the family ${\left(T\left(E|U_{\alpha }\right),T\psi_{\alpha }^{\prime }:T\left(E|U_{\alpha }\right)\to T\left(u_{\alpha }\left(U_{\alpha }\right)\times V\right)=u_{\alpha }\left(U_{\alpha }\right)\times V\times R^{m}\times V\right)}_{Î\pm \in A}$ is the atlas describing the canonical vector bundle structure of $\left(TE,\pi_{E},E\right)$. The transition functions are in turn:

$$\begin{array}{ccc} \left(\psi_{\alpha }\circ \psi_{\beta }^{-1}\right)\left(x,v\right) & = & \left(x,\psi_{\alpha \beta }\left(x\right)v\right)\;\;\;\mathrm{for}\;\;x\in U_{\alpha \beta }\\ \left(u_{\alpha }\circ u_{\beta }^{-1}\right)\left(y\right) & = & u_{\alpha \beta }\left(y\right)\;\;\;\mathrm{for}\;\;y\in u_{\beta }\left(U_{\alpha \beta }\right)\\ \left(\psi_{\alpha }^{\prime }\circ {\left(\psi_{\beta }^{\prime }\right)}^{-1}\right)\left(y,v\right) & = & \left(u_{\alpha \beta }\left(y\right),\psi_{\alpha \beta }\left(u_{\beta }^{-1}\left(y\right)\right)v\right)\\ \left(T\psi_{\alpha }^{\prime }\circ T{\left(\psi_{\beta }^{\prime }\right)}^{-1}\right)\left(y,v;\xi ,w\right) & = & (u_{\alpha \beta }\left(y\right),\psi_{\alpha \beta }\left(u_{\beta }^{-1}\left(y\right)\right)v;\\ & & \;d\left(u_{\alpha \beta }\right)\left(y\right)\xi ,\left(d\left(\psi_{\alpha \beta }\circ u_{\beta }^{-1}\right)\left(y\right)\right)\xi )v+\psi_{\alpha \beta }\left(u_{\beta }^{-1}\left(y\right)\right)w). \end{array}$$

Therefore, we see that for fixed (y,v), the transition functions are linear in $\left(\xi ,w\right)\in R^{m}\times V$. This finding describes the vector bundle structure of the tangent bundle $\left(TE,\pi_{E},E\right)$.

For fixed (y,ξ), the transition functions of TE are also linear in (v,w)∈V×V. This gives a vector bundle structure on (TE,Tp,TM). Its fiber addition will be denoted by $T\left({+}_{E}\right):T\left(E\times_{M}E\right)=TE\times_{TM}TE\to TE$ since it is the tangent mapping of ${+}_{E}$. Its scalar multiplication will be denoted by $T(m_{t}^{E})$.

The space $\Xi \in TE:Tp.\Xi =0\;\;\mathrm{in}\;\;TM={\left(Tp\right)}^{-1}\left(0\right)$ is denoted by VE and is called the vertical bundle over E. The local form of a vertical vector Ξ is $T\psi_{\alpha }^{\prime }.\Xi =\left(y,v;0,w\right)$; thus, the transition function looks like $\left(T\psi_{\alpha }^{\prime }\circ T{\left(\psi_{\beta }^{\prime }\right)}^{-1}\right)\left(y,v;0,w\right)=\left(u_{\alpha \beta }\left(y\right),\psi_{\alpha \beta }\left(u_{\beta }^{-1}\left(y\right)\right)v;0,\psi_{\alpha \beta }\left(u_{\beta }^{-1}\left(y\right)\right)w\right)$.

#### Appendix A.4.7. Vector Bundle Homomorphism/Isomorphism

Let (E,p,M) and (F,q,N) be vector bundles. A vector bundle homomorphism φ:E→F is a fiber-respecting fiber linear smooth mapping (denoted by a diagram):  If φ is invertible, it is called a vector bundle isomorphism.

#### Appendix A.4.8. Connection

A connection on the fiber bundle (E,p,M,S) is a vector-valued one-form (a one-form on a manifold M is a smooth mapping of the total space of the tangent bundle of M to R whose restriction to each fiber is a linear functional on the tangent space, i.e., $\alpha :TM\to R,\;\alpha_{x}{=\alpha |}_{T_{x}M}:T_{x}M\to R$, where $\alpha_{x}$ is linear.) $\Phi \in \Omega^{1}\left(E;VE\right)$ with values in the vertical bundle VE such that Φ∘Φ=Φ and ImΦ=VE; thus, Φ is a projection TE→VE.

Connection defines parallel transport on the fiber bundle, so we can consider it as how fibers are connected over manifolds. If the fiber bundle is a vector bundle as shown in Figure A1, the connection is linear.
